# RNA and Mitochondrial Reprogramming Associated with Azacytidine Treatment in Higher-Risk Myelodysplastic Syndromes: A Pilot Study

**DOI:** 10.3390/cancers18142305

**Published:** 2026-07-17

**Authors:** Theodoros Nikolopoulos, Irene Dereki, Vasiliki Chondrou, Argyri Chroni, Theodora Alexiou, Katerina Athanasopoulou, Eleftherios Bochalis, Theodora Chatzilygeroudi, John Zafeiropoulos, Ilias Georgakopoulos-Soares, Kyriakos Bourikas, Argiris Symeonidis, Argyro Sgourou

**Affiliations:** 1 Biology Laboratory, School of Science and Technology, Hellenic Open University, 263 35 Patras, Greece; nikolopoulos.theodoros@ac.eap.gr (T.N.); ntereki.eirini@ac.eap.gr (I.D.); vchondrou@eap.gr (V.C.); up1065152@ac.upatras.gr (A.C.); alexiou.theodora@ac.eap.gr (T.A.); athanasopoulou.aikaterini@ac.eap.gr (K.A.); 2 School of Health Sciences, Faculty of Medicine, Hematology Division, University of Patras, 265 04 Patras, Greece; 3 Division of Pharmacology and Toxicology, College of Pharmacy, Dell Paediatric Research Institute, The University of Texas at Austin, Austin, TX 78723, USA; ekb2285@eid.utexas.edu (E.B.); 4 Hematology Unit, Second Department of Internal Medicine and Research Unit, National and Kapodistrian University of Athens School of Medicine, Attikon University Hospital, 12462 Athens, Greece; thchatzilygeroudi@gmail.com; 5 Chemistry Laboratory, School of Science and Technology, Hellenic Open University, 263 35 Patras, Greece; jzafeirop@eap.gr (J.Z.)

**Keywords:** higher-risk myelodysplastic syndromes, azacytidine treatment, RNA modifications, miRNA expression signatures, cell pathways, mtDNA depletion

## Abstract

**Aims:** Treatment of higher-risk myelodysplastic syndromes (HR-MDS) with azacytidine (AZA) exerts significant effects on the epigenome, primarily through DNA demethylation and reactivation of epigenetically silenced genes. Beyond this established mechanism, molecular AZA-linked effects are increasingly being recognized. **Materials and methods:** Liquid chromatography combined with mass spectrometry (LC-MS/MS) was employed for the accurate assessment of various RNA and DNA modifications pre- and post-AZA treatment of an HR-MDS cohort (N = 8). Mapping of the AZA treatment-responsive regulatory pathways was performed by miRNA-next generation sequencing (NGS), followed by a multi-layered bioinformatic pipeline, integrating miRNA differential expression, gene set enrichment, and network analyses. The precise number of mitochondrial (mt)DNA copies pre- and post-AZA was evaluated by a digital PCR assay. **Results:** Cell pathways affected by miRNA differential expression patterns pre- and post-AZA treatment discriminated the clinical phenotypes of Responders against Non-Responders to therapy. Intracellular RNA modifications: N6-methyladenosine (m6A), 5-methylcytidine (m5C), N1-methyladenosine (m1A), 2′-O-methylguanosine (Gm) and adenosine-to-inosine (A → I) editing were evaluated for their potential impact in treatment response. Nuclear DNA/mtDNA methylation profiles and mtDNA copy number reduction manifested the mitochondrial features affected by AZA. Our results suggest that neoplastic HSPCs in HR-MDS Responders to AZA adapt by normalizing glycolytic metabolism and enhancing ribosomal activity. The observed reduction of mtDNA content can be associated with improved survival and suppression of malignant progression. Non-Responders, despite experiencing mtDNA depletion, seem unable to coordinate such metabolic reprogramming and remain disadvantaged to AZA therapy.

## Simple Summary

Despite its role as the standard first-line treatment for higher-risk myelodysplastic syndromes (HR-MDS), azacytidine (AZA) is associated with limited response durability and a high incidence of treatment resistance. We have studied the in-depth epigenetic features of eight matched HR MDS samples pre- and post-AZA treatment, and the results have highlighted new molecular targets and revealed permissive molecular mechanisms for response to treatment. HR-MDS Responders exhibit microRNA shifts that modulate critical cell pathways post-AZA treatment. Also, intracellular concentrations of Gm, m6A, 5mC, and m1A RNA modifications were differentiated in Responders against Non-Responders. Evidence of mitochondrial involvement was observed, as the mtDNA copy numbers regressed significantly following completion of AZA treatment.

## 1. Introduction

Myelodysplastic syndromes (MDS) are myeloid neoplasms exerting multiple abnormal hematological phenotypic characteristics, such as ineffective hematopoiesis, disturbed cellular maturation and differentiation, immune abnormalities, peripheral blood cytopenias, and a variable risk of progression to acute myeloid leukemia (AML). Hypomethylating agents (HMAs), principally azacitidine (AZA), are considered to be the standard therapy for higher-risk (HR) MDS, aiming to inhibit clonal expansion and to improve blood counts and survival for HR-MDS patients. However, a substantial fraction of treated patients fail to respond or relapse. Despite the use of AZA for more than 15 years already, the molecular determinants of response and resistance remain incompletely understood and unpredictable [1]. Aberrant DNA methylation is a hallmark of MDS and contributes to transcriptional dysregulation and alteration in hematopoietic stem and progenitor cell (HSPC) differentiation pathways. Genome-wide methylome studies for AZA effects in MDS demonstrate that AZA perturbs CpG (Cytosine followed by Guanosine) methylation at regulatory regions, and that both baseline methylation patterns and early treatment-associated demethylation in HSPCs are predictive of clinical response. Hypomethylated CpGs in Responders prior to AZA treatment are obtained across the HOXA and HOXB clusters of genes, GATA2, EPO, and SNAI1 genes, and significantly overlap with CTCF and STAG2 binding sites. CTCF acts as a chromatin insulator and, along with STAG2, which belongs to the cohesin complex, it facilitates the looping of promoters to distal regulatory regions [2]. When dysregulated, it disrupts the chromatin architecture with unfortunate consequences for the cell. Uniquely hypomethylated CpGs in Responders at an early cycle of AZA treatment (immediately following initial AZA first cycle administration) are enriched for the gene pathways related to myeloid cell and osteoclast differentiation, suggesting that a clinical response to AZA is at least partly attributed to epigenetic reprogramming at the gene loci, required for HSPCs differentiation and production of circulating progeny. On the contrary, the Non-Responder MDS patients post-AZA treatment display different and inconsistent methylation patterns. Moreover, baseline differences in genome methylation patterns indicate that CD34+ stem and progenitor cells are epigenetically primed for AZA response only in a subset of patients [3]. These observations support the use of genome-wide DNA methylation profiling, as both a mechanistic readout of AZA activity and as a source of candidate predictive biomarkers, either for global methylation or for selective CpG sites [4,5].

Apart from DNA methylation, altered intranuclear splicing is a general feature of MDS. Impacted RNA splicing has been suggested to occur as a result of the frequently mutated splicing factors SF3B1 and U2AF1 [6,7], but also from aberrant DNA CpG methylation patterns [8,9,10]. N6-methyladenosine (m6A) RNA modification, acting as a splicing process moderator and with a documented implication in RNA stability and translation [11], has been recognized to play several fundamental roles in hematology and across a wide variety of neoplasias [12]. A considerable number of RNA modifications and the modifying enzymes involved (“writers”, “erasers” and “readers”) influence hematopoietic development and malignant transformation and evolution [13]. Initial studies profiling m6A RNA methylation in AZA treated MDS and AML patients suggested the treatment-associated shifts in RNA modification patterns that correlate with distinct transcriptional programs. Elevated m6A and m6A methyltransferase 14 (METTL14) expression has been reported to represent a functional link in HR-MDS pathophysiology, through activation of the PI3K–AKT signaling pathway [14]. Moreover, other RNA-modified nucleosides, such as N1-methyladenosine (m1A), 5-methylcytosine (m5C), and adenosine-to-inosine (A → I) editing, have emerged as critical events for HSPCs maintenance and homeostasis [15,16,17]. Thus, mapping RNA modifications provides complementary functional information to aberrant epigenome status [18].

MicroRNAs (miRNAs) form an additional regulatory layer that control and shape gene regulatory networks in MDS. Multiple studies have reported the various miRNA expression signatures that distinguish Responders from Non-Responders to AZA, and specific miRNAs have been proposed as circulating or cellular potential biomarkers of response. MiRNAs mediate the post-transcriptional control of gene networks and are themselves regulated by DNA methylation and RNA modifications; therefore, integrating miRNA profiling with other layers can reveal coherent regulatory axes relevant to HMA therapy [19,20].

Finally, mitochondrial biology, as assessed by the mtDNA methylation levels and copy numbers per cell can be directly correlated with mitochondrial dysfunction and has emerging relevance in MDS. Studies have documented frequent but modest mtDNA mutations in bone marrow cells from MDS patients [21,22], and with unclear functional importance. AZA treatment has been shown to reduce overall DNA methylation levels, including mtDNA in adipose tissue, and to alter oxidative stress pathways, suggesting that mitochondrial regulation is linked to epigenetic remodeling [23]. The increased mtDNA copy numbers in CD34+ marrow cells, despite a generally decreased mitochondrial gene expression pattern, have also been reported [24]. Given the biochemical coupling between mitochondrial metabolism and chromatin, the quantification of mtDNA copy numbers, alongside nuclear epigenetic and epitranscriptomic layers can reveal potential crosstalk that impacts AZA response [25].

We therefore have conducted a multi-layered, integrative epigenetic study of AZA effects in the matched pre- and post-treatment HR-MDS patients’ samples to reveal the coordinated regulatory changes and to nominate the biomarkers of response to therapy. We aimed to address the hypothesis that AZA treatment induces coordinated remodeling across nuclear and mitochondrial regulatory layers in MDS; specifically, (i) AZA treatment-associated changes in global nuclear (n)DNA and mitochondrial (mt)DNA methylation levels, (ii) alterations in RNA modifications affected by AZA treatment, including m6A, m5C, m1A, Gm, and A → I editing, causative for variations in HSPC self-maintenance state and fate, (iii) shifts in miRNA networks that reinforce or attenuate transcriptional programs and cell pathways post-AZA treatment, and (iv) directional changes in mtDNA copy number, reflecting altered mitochondrial biogenesis or selection of subclones. Our study indicated integrated multi-epigenetic signatures, combined with mtDNA copy number variations. Our results highlight novel underlying molecular changes related to AZA treatment and discriminate clinical Responders from Non-Responders more efficiently than any single layer alone [20,26].

## 2. Materials and Methods

### 2.1. Patient Recruitment–AZA Treatment Monitoring-Exclusion Criteria

Myelodysplastic Syndromes (MDS) patients were diagnosed, followed, and treated in the Hematology Division of Rion Patras University Hospital. Diagnosis and classification were performed according to the World Health Organization (WHO) criteria. Only chemotherapy-naive patients with higher risk (HR) MDS were recruited in the present study, whereas patients with chronic myelomonocytic leukemia (CMML) or chemo/radiotherapy-associated secondary MDS or AML were excluded. The study protocol concerning scientific research questions in HR-MDS using clinical data and biological samples was approved by the Rion University Hospital Ethics Committee (approval decision number 33807/approval date 24 December 2020). Written informed consent was obtained from all patients, in accordance with the Declaration of Helsinki.

The HR-MDS patients received azacytidine (AZA) subcutaneously by standard scheduling (75 mg/m^2^  ×  7 consecutive days, 28-day cycle). Patients who progressed to AML before the completion of the initial 6 cycles of treatment or who died by any cause, occurring after randomization and through the 6 initial AZA treatment cycles, were also excluded from the study (patient NR4, who has been included in the study, progressed to AML soon after the completion of the 6th cycle of AZA treatment). In monitoring the AZA response, the marrow complete response (mCR) was defined according to the International Working Group response (IWG) 2006 criteria as that containing myeloblasts  ≤  5% and decreased  ≥  50% over pre-treatment at the first evaluation of response (5–7 cycles of AZA) [27]. Hematological improvement (HI) was defined according to the revised IWG 2018 hematological response criteria [28]. Eight (8) HR-MDS patients who successfully completed 6 cycles of AZA treatment were monitored and included in the present study, further categorized as AZA Responders (R) or AZA Non-Responders (NR). Matched pre- and post-AZA bone marrow specimens were collected, and nuclear and mitochondrial DNA and total RNA samples were isolated from the bone marrow mononuclear cells (BMMCs) and tested for quality and purity and then subjected to downstream molecular analysis. Table 1 presents the HR-MDS patients’ hematological records.

### 2.2. MiRNA Profiling by Next Generation Sequencing-Data Processing and Interpretation

Total RNA, including microRNA species, was purified from the HR-MDS patients’ BMMCs pre- and post-AZA treatment (Table 1). RNA quality and integrity were assessed using the QIAxcel RNA QC Kit v2.0 (Qiagen, GmbH, Germany, 929104) on the QIAxcel^®^ Advanced System following the manufacturer’s instructions. All RNA samples showed high integrity scores (RIS), ranging from 8.5 to 9.5, except for sample R4’s pre-AZA treatment (low RIS < 4.5). The miRNA library from this sample twice failed to be generated due to the poor quality of isolated RNA; therefore, this patient’s sample was excluded from next generation sequencing (NGS). Libraries were constructed (QIAseq miRNA library kit, QIAGEN GmbH, Hilden, Germany) and RNA sequencing reactions were performed in Illumina iSeq 100 instrument (Illumina, Inc., San Diego, CA, USA), as previously described [29]. The NGS parameters were single-ended mode with 75 bp sequence length, with total reads ranging from 0.6 to 1.5 million per sample (Supplementary Table S1). Raw sequencing data (FASTQ files) were processed using a custom BASH pipeline to generate expression counts. Initial quality control was performed using FastQC (v0.12.1) [30]. Unique molecular identifiers (UMIs) were extracted from the reads using UMI-tools (v1.1.6), to be later used for deduplicating counts with a regular expression pattern (RegEx). The term, +(?P<discard_1>AACTGTAGGCACCATCAAT){s<=2}(?P<umi_1>.{12})(?P<discard_2>.+), that searches each sequence for the 3′ adapter (AACTGTAGGCACCATCAAT), extracts the following 12 bases and stores the sequences as UMIs, and discards any nucleotides after that. Adapter sequences were trimmed and reads were quality-filtered (minimum length 18 bp, quality cutoff 20) using Cutadapt (v5.2) [31].

#### 2.2.1. Alignment and Quantification

Processed reads were aligned to the human mature miRNA reference index (based on miRbase [32]) using BWA.aln (v0.7.19) [33] with strict parameters (-n 1 -o 0 -e 0 -l 8 -k 0) to allow only high-quality alignments with zero mismatches in the seed sequence. SAM files were converted to the BAM format and sorted using SAMtools (v1.21) [34]. PCR duplicates were computationally removed from the aligned BAM files using UMI-tools dedup, utilizing the extracted UMI information to retain unique biological reads. Final count data were generated by quantifying the deduplicated reads mapped to each mature miRNA using samtools idxstats.

#### 2.2.2. Differential Expression Analysis of miRNA

Raw miRNA count data were analyzed using the DESeq2 R package (v1.44.0) [35] to identify the differential expression associated with AZA response. Low-abundance miRNAs were filtered out prior to normalization, and only those with at least 10 counts in at least 2 samples were retained. To dissect the effect of treatment response over time, we utilized a generalized linear model with the interaction design ~ response + time + response:time. The interaction term identifies the miRNAs showing differential temporal changes in Responders compared to Non-Responders.

#### 2.2.3. Gene Set Enrichment Analysis

Since individual miRNAs failed to meet the specified significance after multiple testing correction, we performed the gene set enrichment analysis (GSEA) to identify modest coordinated pathway alterations. A ranked list of miRNAs was generated for the GSEA by calculating a signed statistic for each miRNA: sign(log_2_FoldChange) × −log10(*p*-value). Infinite values resulting from *p*-values of zero were capped at 1000 to maintain ranking stability.

The GSEA was performed using the fgsea package (v1.34.2) [36]. Pre-processed miRNA gene sets were obtained from the miRNA Enrichment Analysis and Annotation (miEAA 2.0) database [37]. We queried four validated miRNA-set collections: Pathways (miRWalk), Gene Ontology (miRWalk), Target genes (miRTarBase, experimentally validated targets), and Biological process annotations (miRPathDB). Gene sets were filtered to include those with a minimum size of 10–15 miRNAs and a maximum of 500. Pathways were ranked by their normalized enrichment score (NES), which accounts for differences in gene set size, and those with a Benjamini–Hochberg adjusted *p*-value (padj) < 0.05 were considered to be significantly enriched.

#### 2.2.4. Integrated Network Analysis

To visualize the regulatory architecture of the drug responses, we constructed an integrated miRNA-pathway network using the ggraph (v2.2.2) and tidygraph (v1.3.1) packages. We selected the top significant biological themes (Pathways, Gene Ontology, Target Genes, and Biological Processes). Edges were defined by the subset of miRNAs driving the enrichment of each pathway. In the final network visualization, biological themes were depicted as square nodes and miRNAs were depicted as circular nodes. miRNA nodes were colored according to their log2FoldChange in the interaction analysis, highlighting the specific upregulation or downregulation of regulatory hubs in Responders versus Non-Responders over AZA treatment.

### 2.3. Detection of RNA Modifications by Liquid Chromatography Combined with Mass Spectrometry (LC-MS/MS)

#### 2.3.1. Methodology

Liquid Chromatography (LC) with a triple quadrupole (QqQ) mass spectrometer (LCMS-8050 system, Shimadzu, Kyoto, Japan) was utilized to analyze the total RNA samples from the HR-MDS patients pre- and post-HMA treatment (Table 1). AZA treatment-associated changes in N6-methyladenosine (m6A), 5-methylcytidine (m5C), N1-methyladenosine (m1A), 2′-O-methylguanosine (Gm), and adenosine-to-inosine (A → I) editing (inosine) RNA modifications were assessed. Prior to the LC-MS/MS analysis, 300 ng of each RNA sample was digested and dephosphorylated by a Nucleoside Digestion Mix (NEB#M0649) to generate RNA nucleosides for further quantitative analyses, following the manufacturer’s instructions.

The LC-MS/MS system features a heated electrospray ionization (ESI) system and multiple reaction monitoring (MRM) capabilities with high sensitivity and high speed. The column oven was set at 35 °C. A Shim-pack Scepter C18 column (4.6 mm × 100 mm, 5 µm, Shimadzu) with a 4.6 mm pre-column was used for the separation of RNA nucleosides, carrying modifications for analysis. The mobile phase was passed through the column by gradient elution with acidified H_2_O (0.1% HCOOH) (solvent A) and acidified acetonitrile (ACN-0.1%HCOOH) (solvent B) [38]. The flow rate was set at 0.2 mL/min with the solvent ratio starting at 95% A/5% B, and reaching 35% A/65% B at 15 min. The total analysis time was 20 min and the injection volume was 10 µL. Mass spectrometry detection was performed under the positive electrospray ionization (ESI) mode. The RNA modifications were monitored by multiple reaction monitoring (MRM) modes using the mass transitions (precursor ions → product ions) of m5C (258 → 126), m6A (282.1 → 150), Gm (298 → 152), inosine (269 → 137), and m1A (282 → 150) [39]. Calibration curves of m5C, m6A, Gm, inosine, and m1A were constructed by plotting the peak area versus concentration, respectively, based on data obtained from the LC-ESI-MS/MS analysis. Linearity was within the concentration range 0.5–20 ppb for m5C, 0.2–10 ppb for m6A, 0.5–100 ppb for Gm, 5–500 ppb for inosine, and 0.1–10 ppb for m1A, with a coefficient of determination (R2) greater than 0.99.

#### 2.3.2. NGS and RNA Modification Data Processing and Statistical Analysis

Statistical analyses and data visualization were performed using R (v2024.12.1+563). Prior to analysis, two outlier values post-treatment, specifically patient R4 for modification of m5C and patient NR2 for modification of m6A, were excluded from the analysis, as they were technically reproducible but markedly outside the distribution of the remaining observations (>30-fold and >3-fold deviation from the mean, respectively). Only those patients with paired samples (both, pre- and post-treatment timepoints) were retained, to ensure consistent comparisons. The change in modification levels (Delta) was calculated for each patient as the difference between the post-treatment and pre-treatment levels (Delta = Post − Pre). Non-parametric statistical tests were utilized; Wilcoxon signed-rank test was employed to assess any significant changes in the modification levels over time (“Pre” vs. “Post”) across the entire cohort, and the Mann–Whitney U test (Wilcoxon rank-sum test) was used to compare the calculated Δ values between Responders and Non-Responders and to evaluate the treatment outcome.

Data were visualized using boxplots to illustrate the distribution of changes between the response groups. Statistical tests were two-sided to account for both the increased and decreased values. A *p*-value of <0.05 was considered to be statistically significant.

### 2.4. 5′ Methyl-2′ Deoxycytidine (5mdC) Levels Detection in Nuclear and Mitochondrial DNA

A total of 300 ng from nuclear DNA (nDNA) and mitochondrial DNA (mtDNA) fractions from the HR-MDS patient cohort pre- and post-AZA treatment were separately hydrolyzed into nucleosides. Prior reaction for each DNA fraction was cleaned from any RNA residuals by performing an RNase reaction. Single nucleosides were then separated and quantified by LC-MS/MS methodology, as previously described [5]. The calibration curve of 5mdC was constructed by plotting the peak area ratios of 5mdC/dG versus the molar ratio of 5mdC/10^2^dG (accepted R^2^ > 0.99). The modified form of deoxy-cytosine 5mdC, indicating the methylation levels on either nDNA or mtDNA sample, was evaluated from the mean values derived from two–three independent runs.

Statistical analyses and data visualization were performed using R (v2024.12.1+563) for 5mdC DNA modification, as described for RNA modifications.

### 2.5. mtDNA Copy Number Assessment by Digital PCR

DNA samples from the HR-MDS patients pre- and post-AZA treatment (Table 1) were initially diluted and subjected to mechanical fragmentation by repeated passage through a 21G syringe needle. DNA concentration and purity were estimated spectrophotometrically, by measuring absorbance at 230, 260, and 280 nm. Serial dilutions were performed to obtain the final DNA concentrations of 50 ng and 0.5 ng with high accuracy. Digital PCR (dPCR) assays were conducted using primer sets, targeting the mitochondrial DNA (mtDNA) regions encoding the cytochrome b (MT-CYB) (Forward primer: AAAGACGCCCTCGGCTTACT, Reverse primer: TTTGTTAGGGACGGATCGGAG), as well as an intronic region of a nuclear DNA (nDNA) reference gene, the glyceraldehyde-3-phosphate dehydrogenase (GAPDH) (Forward primer: CGGGTCTTTGCAGTCGTATG, Reverse primer: CTGTTTCTGGGGACTAGGGG). Reactions were prepared using the SYBR Green Absolute Q DNA Digital PCR Master Mix (4×), which was constituted in-house immediately prior to use by combining the Applied Biosystems™ Absolute Q DNA Digital PCR Master Mix (5×) with SYBR Green I Nucleic Acid Gel Stain (25×) (Invitrogen™, Carlsbad, CA, USA), according to the manufacturer’s formulation guidelines. For nDNA quantification, reactions contained 50 ng of the template DNA and primers at a final concentration of 200 nM. For mtDNA quantification, reactions contained 0.5 ng of the template DNA and primers at a final concentration of 300 nM. All reactions were prepared in a final volume of 10 μL. A non-template control was included in each run.

Following the reaction setup, 9 μL of each reaction mixture was loaded onto a QuantStudio™ Absolute Q™ MAP16 Plate (QuantStudio™ Absolute Q™ MAP16 Plate Kit, Applied Biosystems™) according to the manufacturer’s instructions. Digital PCR was performed on the Applied Biosystems™ QuantStudio™ Absolute Q™ Digital PCR System using the following thermal cycling protocol: pre-heating at 96 °C for 10 min, followed by 45 cycles of denaturation at 96 °C for 5 s and annealing/extension at 62 °C for 20 s.

The mitochondrial DNA copy number per cell (mtCN) was calculated using the following equation:

(1) mtCN = 2 × DF × [mtDNA]/[nDNA],

where DF represents the dilution factor, [mtDNA] and [nDNA] correspond to the measured copies of the mitochondrial and nuclear targets per μL of dPCR reaction, respectively, as previously described [40].

#### Data Processing and Statistical Analysis

Data analysis and visualization were performed using R (v2024.12.1+563). The patient trajectories were visualized using paired spaghetti plots. The difference in mtDNA levels before and after AZA treatment was quantified by calculating the change in the mtDNA copy number for each patient (Delta) as follows:

(2) Δ = mtCNPost − mtCNPre

The Delta values were plotted using boxplots displaying the calculated difference, stratified by response status.

Non-parametric statistical tests were employed for all comparisons, due to the limited sample size (n = 4 per group). The Wilcoxon signed-rank test (paired) was used to assess the change in mtDNA levels between “Pre” and “Post” timepoints across the cohort. The Mann–Whitney U test (Wilcoxon rank-sum test) was used to compare the distribution of Δ values between the Responder and Non-Responder groups. The *p*-values were two-sided. Significance was defined as *p* < 0.05.

## 3. Results

### 3.1. Differential Modulation of miRNA Networks Discriminates AZA Responder Against Non-Responder HR-MDS Patients

MiRNA libraries prepared from the HR-MDS patient bone marrow samples, collected before and after completion of six cycles of AZA therapy, were subjected to next-generation sequencing (NGS). The NGS data have been deposited in NCBI’s Gene Expression Omnibus and are accessible through GEO Series accession number GSE315656 “https://www.ncbi.nlm.nih.gov/geo/query/acc.cgi?acc=GSE315656” (accessed on 5 January 2026). The patients were further stratified into Responders (n = 3) and Non-Responders (n = 4), enabling comparisons of the differentially expressed miRNAs between the patient groups (Table 1). Patient R4 was excluded from the NGS due to the unsuccessful generation of an miRNA library (low RIS sample).

**Figure 1:**
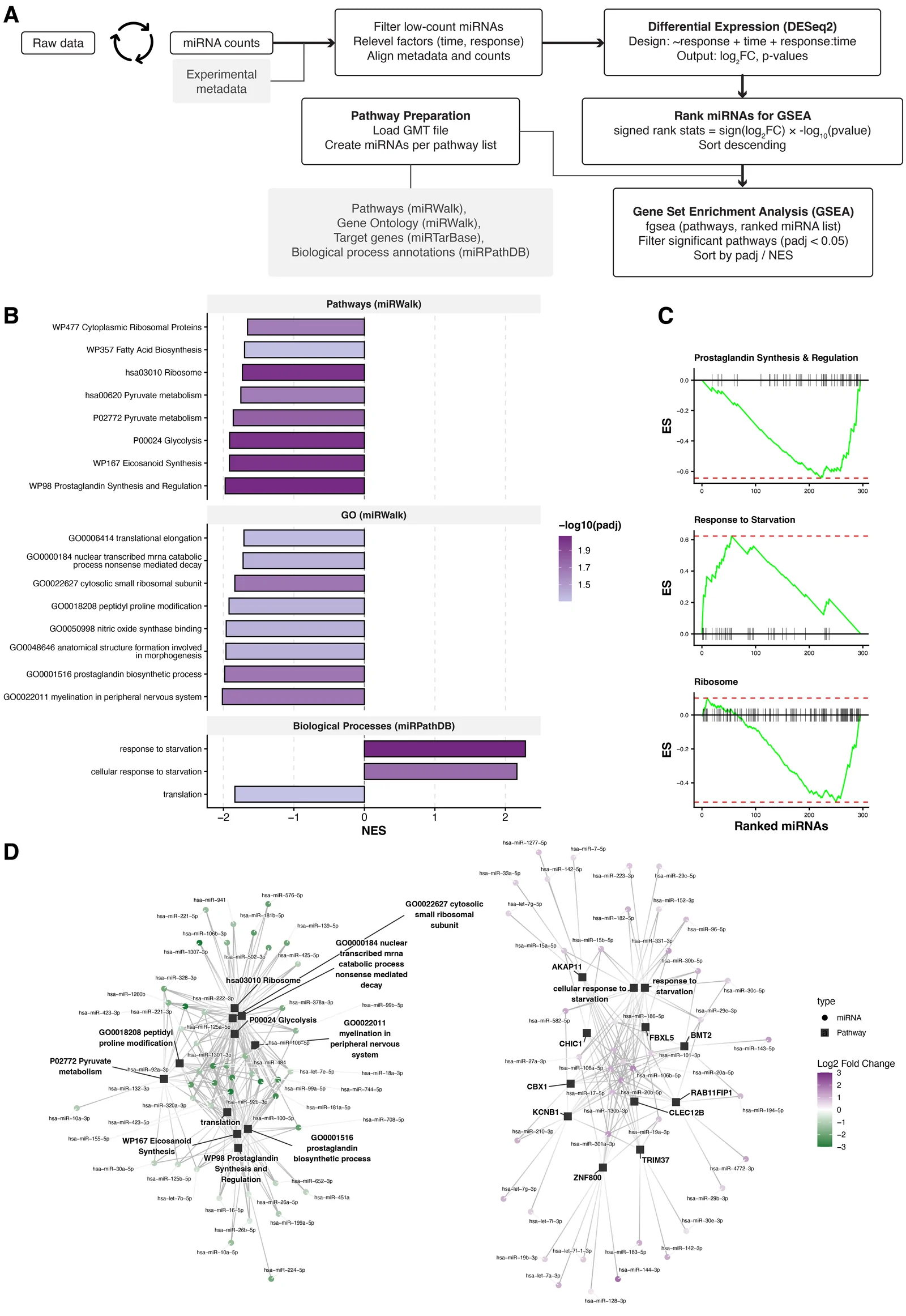
Integrated bioinformatic analysis of miRNA signatures in AZA Response. (**A**) Schematic overview of the computational pipeline. Raw miRNA sequencing reads were processed, filtered, and aligned to the human mature miRNA reference index. Differential expressions were assessed using DESeq2 with a time-dependent interaction design to isolate any miRNA changes specific to the AZA response. A ranked list of miRNAs was used for the GSEA against multiple validated databases (miRWalk, miRPathDB, miRTarBase). (**B**) Functional Landscape of AZA Response. Bar plot summarizing the top significantly enriched biological themes, differentiating Responders from Non-Responders. The x-axis displays the normalized enrichment score; positive values indicate the pathways upregulated in Responders over time, compared to Non-Responders, while negative values indicate downregulation. The color intensity of each bar corresponds to the statistical significance (−log10 adjusted *p*-value). (**C**) Representative Enrichment Profiles. Gene set enrichment plots for three key driver pathways identified in the analysis: Prostaglandin Synthesis and Regulation (**top**), Response to Starvation (**middle**), and Ribosomal Proteins (**bottom**). The green curve traces the running enrichment score across the ranked list of miRNAs, while the vertical black lines (barcode) mark the positions of individual miRNAs belonging to each gene set. (**D**) Integrated Regulatory Network. A network visualization connecting significant biological themes (square nodes) to their regulatory miRNAs (circular nodes). Edges represent the “leading edge” subset of miRNAs, driving the enrichment of each pathway. miRNA nodes are colored according to their log_2_fold change (interaction term), where purple indicates upregulation and green indicates downregulation in Responders relative to Non-Responders over the course of treatment.

Initial differential expression analysis (DEA) using the statistical threshold of FDR < 0.05, did not identify individual miRNAs with significant upregulation or downregulation in Responders, compared to Non-Responders, during the AZA treatment (Supplementary Figure S1, Supplementary Table S2). The absence of significance among single miRNAs suggests that the therapeutic response to AZA might more likely be mediated by coordinated changes in gene regulatory networks, rather than by a limited number of isolated regulatory factors. We applied the GSEA to capture these functional changes of collective targets, using the ranked differential expression statistics (Figure 1A). This approach revealed two distinct regulatory programs, characterized by opposing trends in metabolic reactivation and stress suppression, separating the AZA effect and response outcome.

#### 3.1.1. De-Repression of Biosynthetic and Metabolic Pathways in Responders

The first major regulatory module was defined by miRNAs with significant negative interaction coefficients (downregulated in Responders relative to Non-Responders during AZA treatment). Given the repressive nature of miRNAs in general, the depletion of these miRNAs implies a “de-repression” or reactivation of their downstream targets. The GSEA using miRWalk [41] pathways and Gene Ontology (GO) databases revealed the consistent enrichment of pathways essential for cellular metabolism and protein synthesis in this module (Figure 1B). Specifically, we observed significant enrichment for “P00024_Glycolysis” (NES = −1.91, P_adj_ = 0.0097) and “P02772_Pyruvate_metabolism” (NES = −1.86, P_adj_ = 0.0160), suggesting a restoration of bioenergetic capacity in the responding patients (Supplementary Figure S2). Furthermore, this module showed a strong signature for translational machinery, indicated by the enrichment of “GO0022627_cytosolic_small_ribosomal_subunit” (NES = −1.84, P_adj_ = 0.0207), “hsa03010_Ribosome” (NES = −1.73, P_adj_ = 0.0097), and “WP477_Cytoplasmic_Ribosomal_Proteins” (NES = −1.66, P_adj_ = 0.0224) (Figure 1B,C). Additionally, we identified a significant association with inflammatory signaling precursors, specifically “WP98_Prostaglandin_Synthesis_and_Regulation” (NES = −1.97, P_adj_ = 0.0087), and “GO0001516_prostaglandin_biosynthetic_process” (NES = −1.98, P_adj_ = 0.0206) (Supplementary Figure S3). The downregulation of miRNAs targeting these pathways in Responders suggests an environment where restoration of hematopoietic differentiation and activity can take place (Figure 1D).

#### 3.1.2. Active Suppression of Starvation and Stress Responses

The second regulatory module was defined by miRNAs with positive interaction coefficients (maintained or upregulated in Responders relative to Non-Responders during AZA treatment). This profile revealed upregulation of miRNAs targeting stress–response signaling processes, suggesting that Responders are more likely to suppress these pathways via miRNA-mediated regulation. The top enriched biological processes from the GSEA, using mirPathDB [42] and the Biological Processes GO dataset included “response_to_starvation” (NES = 2.28, P_adj_ = 0.0086) and “cellular_response_to_starvation” (NES = 2.16, P_adj_ = 0.0182) (Figure 1B,C, Supplementary Figure S4). To validate these functional associations, we examined the enrichment of validated miRNA target genes using the miRTarBase dataset. The analysis confirmed that miRNAs targeting stress-associated genes were significantly enriched, with top hits including *KCNB1* (NES = 2.36, P_adj_ = 0.0117), a potassium channel linked to oxidative stress regulation, and *FBXL5* (NES = 2.22, P_adj_ = 0.0126), a key regulator of iron homeostasis and oxygen sensing. Other significant targets included *TRIM37* (NES = 2.32, P_adj_ = 0.0117) and *CLEC12B* (NES = 2.21, P_adj_ = 0.0142) (Supplementary Figure S5). These data support a model in which a deactivation of stress-induced signals takes place during AZA treatment through the maintenance or upregulation of miRNAs in Responders that are not seen in non-Responders (Figure 1D, Supplementary Figures S6 and S7).

### 3.2. AZA Treatment-Associated Mild Shifts of Global m5C, m6A, m1A, Gm, and Inosine RNA Modifications

**Figure 2:**
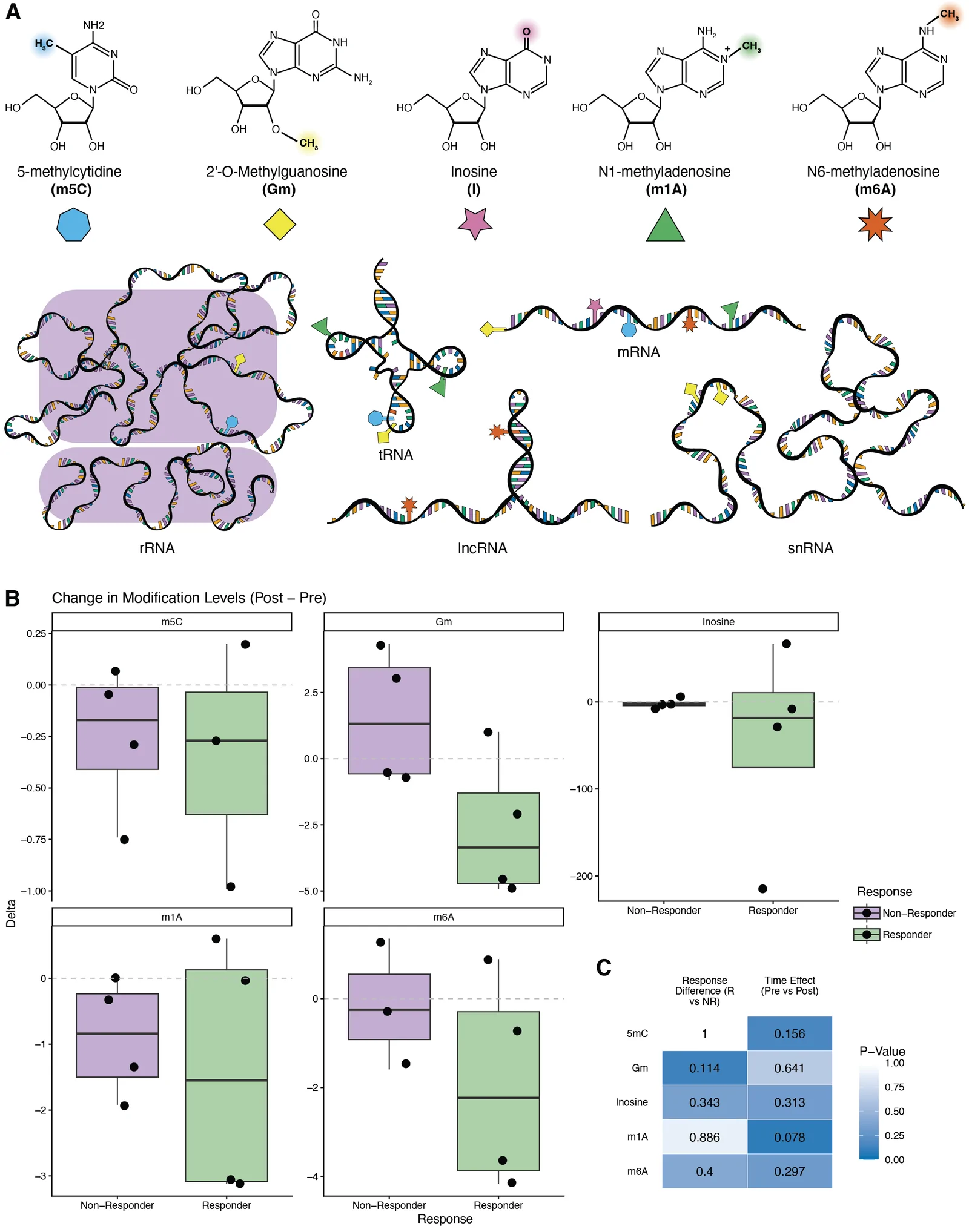
Changes in global RNA modification levels in the HR-MDS patients treated with AZA. (**A**) Chemical structures (**top**) and distribution (**bottom**) of RNA molecules of five modified nucleosides. m5C occurs in tRNA, rRNA, and mRNA, facilitating stability and oxidative stress responses. Gm is abundant in snRNA, rRNA, and tRNA, and appears in the mRNA 5′ cap. Inosine marks A-to-I edited sites across mRNAs. m1A targets tRNA and rRNA to support secondary structure and translation initiation. m6A regulates splicing, stability, and export in eukaryotic mRNAs and lncRNAs. (**B**) Boxplots of differential changes in modification levels. The change in modification levels (Δ = post-treatment with AZA–pre-treatment with AZA) is shown for Non-Responders (purple, n = 4) and Responders (green, n = 4). Individual patient data points are overlaid on boxplots representing the median and interquartile range. The dashed line (y = 0) indicates no change; values below the line indicate a decrease in the modification levels following treatment. (**C**) Heatmap of the statistical significance of observed changes. A summary of *p*-values derived from non-parametric tests. Response Difference (Left Column): Significance of the difference in Δ values between Responders and Non-Responders (Mann–Whitney U test). Time Effect (Right Column): Significance of the change from pre- to post-treatment across all patients (Wilcoxon signed-rank test). Darker blue in the heatmap indicates higher statistical significance.

Five of the most prevalent and well-characterized chemically modified RNA nucleosides were analyzed among the HR-MDS cohort participating in our study. m6A is a post-transcriptional modification of eukaryotic messenger (m)RNAs and long non-coding RNAs, recognized by several specific protein readers and preserving critical functions in various biological processes, such as splicing, mRNA stability and export, and regulation of translation, among others [43]. m5C and m1A are also two modifications occurring along eukaryotic RNAs. m5C is commonly found in transfer (t)RNA, specific sites of mRNA, and ribosomal (r)RNA, and has been proven to be necessary for mRNA stability, nuclear-cytoplasmic shuttling, translation, and oxidative stress response [44]. m1A is predominantly localized to tRNA, mRNA, and rRNA, has been suggested to facilitate the RNA secondary structure and translation initiation, and acts as a potential regulator in response to serum starvation and heat shock conditions [45,46,47]. Gm is highly abundant in non-coding RNAs, including rRNA and tRNA. Its presence has been documented in small nuclear (sn) RNAs, which are part of the splicing machinery, as well as at the 5′ cap and internal sites of eukaryotic mRNAs [48]. A → I editing is a widespread post-transcriptional modification found across multiple RNA classes (Figure 2A). A foundational study mapped A → I edited sites across the human genome and showed that inosine is very common in mRNA, but also in non-coding RNAs and in transcripts, deriving from repetitive elements [49].

Limitations deriving from the patients’ sample size enabled only total RNA extraction, with no further isolation of RNA sub-classes (mRNA, tRNA, rRNA, lncRNA, etc.). Also, the endogenous cell levels of the studied RNA modifications were unknown. Total RNA isolated from the HR-MDS patients pre- and post-AZA treatment was digested and dephosphorylated to generate single nucleosides for the LC-MS/MS analysis. We initially designed standard curves to span a wide dynamic range of concentrations, as well as multi-standards encompassing several combinations of the RNA modified nucleosides to avoid any discrepancies due to elution times and interactions between different, chemically modified, and unmodified RNA nucleosides. A serial dilution series covering several orders of magnitude were prepared to compensate for the unknown intracellular modified RNA molecules’ concentration. To control variability in the sample preparation and instrument response, all calibration standards were also spiked into this matrix to mimic ion suppression and recovery effects. The spiking of standards by default was additionally utilized to track potential degradation of the chemically fragile modified RNA nucleosides. Total RNA from the human erythroleukemia cell line K562 was also digested and processed the same way as a sample control, to test for the RNA extraction method. K562 RNA samples were simultaneously eluted with a standard method with Trizol® (see the Section 2) or by using filter cleaning kits to obtain optimal isolation results for the unstable RNA modifications.

The standard range was adjusted to bracket the observed cellular concentration from the human Κ562 cell line. LC-MS/MS detection was optimized by linear response confirmation across the concentration range of 0.5–20 ppb for m5C, 0.2–10 ppb for m6A, 0.5–100 ppb for Gm, 5–500 ppb for inosine, and 0.1–10 ppb for m1A. The coefficient of determination was greater than 0.99 (R^2^ > 0.99). RNA extraction with Trizol® was the method of choice due to the apparent difference in the RNA modifications’ capability of maintenance and successful detection.

A double run analysis was performed for each of the biological HR-MDS samples, including standards, to ensure reproducibility across runs. LOD and LOQ were estimated from the calibration curves, and intra-day precision was assessed from same-day duplicate injections of each sample and expressed as %RSD (Supplementary Tables S3–S5). Averages from each sample’s values were fitted to the standard curve and the concentration of each RNA modification was accurately extracted. Delta values (Post–Pre) were calculated and plotted stratified by the response status (Figure 2B). Non-Responders exhibited a mild decrease in m5C, m6A, and m1A levels, while Responders showed contradictory results with inconsistency between the patients. However, Responders showed a clear tendency of decreased Gm (*p* = 0.114), against Non-Responders, who showed an increase. Two outlier values (patient R4, post-AZA treatment for m5C and patient NR2, post-AZA treatment for m6A, Table 1) were excluded from the analysis due to biologically implausible values. Inosine intracellular levels remained almost unaffected by AZA treatment.

Despite the absence of statistical significance, the observed reduction mainly in Gm-modified RNA nucleosides suggests a potential biological trend that warrants further investigation into a greater patient sample and from variable isolated RNA classes.

### 3.3. Global Nuclear DNA Methylation Levels Are Reduced Post-AZA Treatment

**Figure 3:**
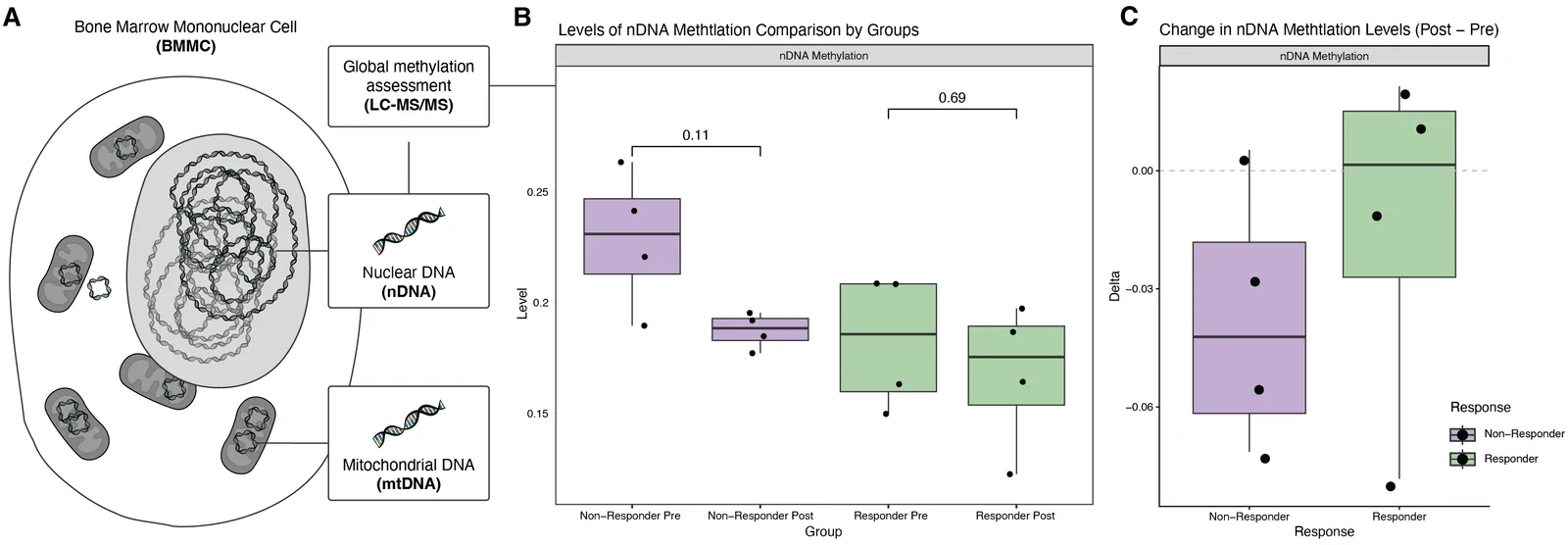
Methylation levels of nDNA. (**A**) nDNA and copies of mtDNA located at the nucleus and mitochondria, respectively, of BMMCs. (**B**) Changes in nuclear DNA (nDNA) methylation levels were evaluated within the same subjects over time (pre-treatment vs. post-treatment) by a two-tailed Wilcoxon signed-rank test (paired). (**C**) Boxplots of differential changes in nDNA modification levels (Δ = post-treatment with AZA–pre-treatment with AZA) are shown for Non-Responders (purple, n = 4) and Responders (green, n = 4). Individual patient data points are overlaid on boxplots representing the median and interquartile range.

The LC-MS/MS analysis was performed to evaluate any changes in 5mdC DNA modification among the HR-MDS patient samples collected before and after AZA treatment. Both nDNA and mtDNA fractions from each patient sample were subjected to this kind of methylation analysis. Very limited experimental evidence supports that the treatment of cancer with the hypomethylating agent AZA can affect mtDNA methylation or mitochondrial epigenetic regulation [50]. Mammalian mtDNA methylation levels have been previously evaluated between 0.2% and 0.7%; however, the relevant studies have emphasized the need for technical advancements to produce reliable results by reducing potential false positive mtDNA 5mdC signals [51]. In our study, mtDNA revealed insignificant results in all relevant comparisons, indicating a stable level of 0.2 (5mdC/102dG) across the HR-MDS cohort, either the pre- or post-AZA therapy time points. On the other hand, although decreased values in the signal intensity of nDNA were observed at the post-AZA treatment time point more clearly in the Non-Responder group of patients (0.23 pre-AZA vs. 0.19 post-AZA), the statistical analysis indicated that those differences were not significant (*p* = 0.11). Responders displayed minimal levels of methylation reduction (0.19 pre-AZA vs. 0.17 post-AZA, *p* = 0.69) (Figure 3A–C).

The LC-MS/MS method demonstrated stable analytical performance across all analyzed samples, with consistent retention times and reproducible peak areas. Overall, these results suggest that, within the sensitivity and variability of the method, AZA treatment did not produce statistically significant changes in the measured levels of 5mdC DNA marker.

### 3.4. AZA Therapy Induces mtDNA Copy Number Regression

**Figure 4:**
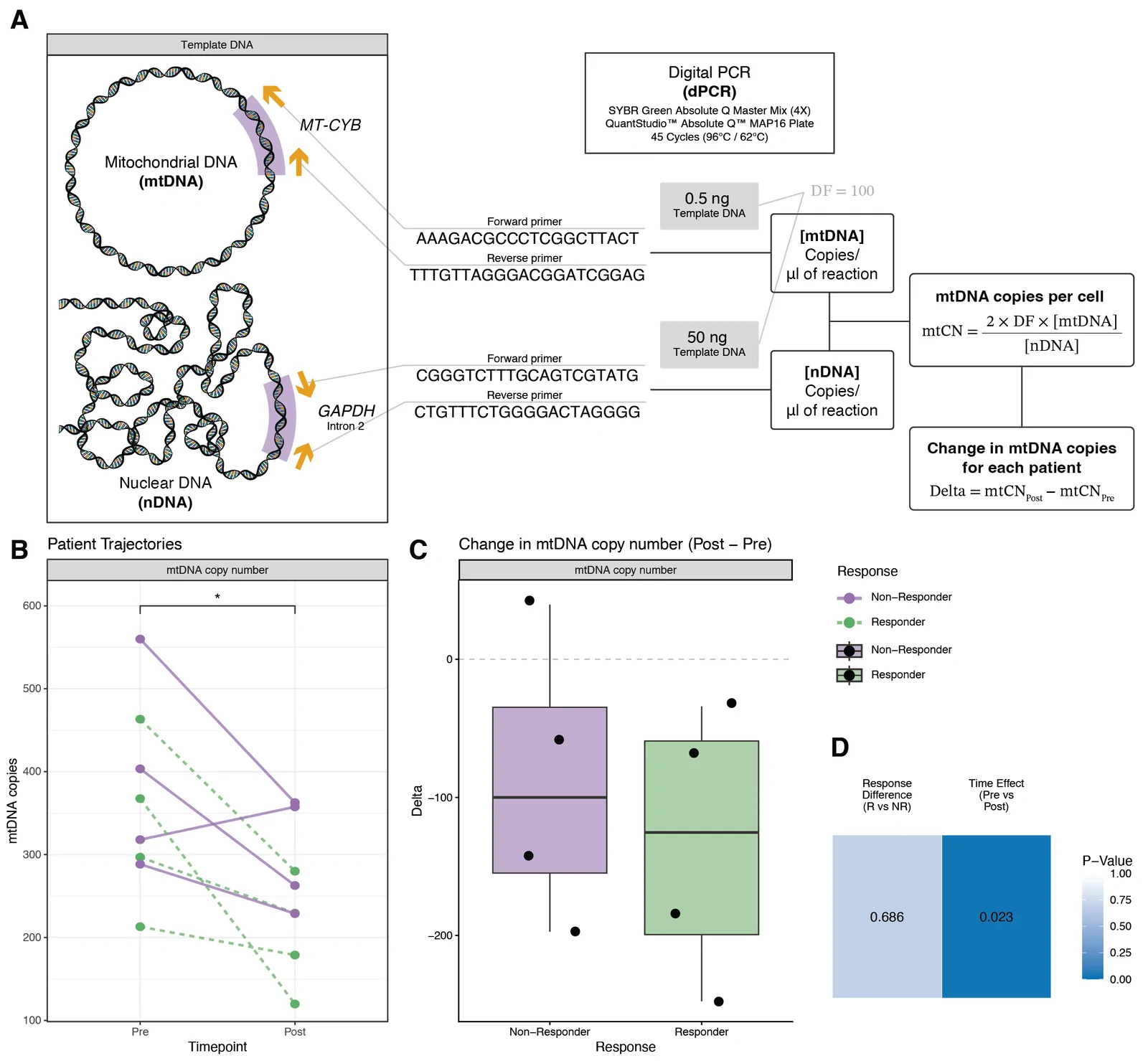
Absolute quantification of the mtDNA copy number in the HR-MDS patients treated with AZA. (**A**) Schematic of the Digital PCR (dPCR) workflow for the mtDNA trend indicates a decrease in the copy number over time quantification. The assay targets the mitochondrial gene MT-CYB and the nuclear gene GAPDH (Intron 2) to calculate the ratio of mitochondrial to nuclear DNA. The diagram details the specific forward and reverse primer sequences used to input template amounts (0.5 ng for mtDNA and 50 ng for nDNA), and the calculation formula used to determine the mtDNA copies per cell (mtCN) and the Delta value for each patient. (**B**) Spaghetti plot illustrating the change in mtDNA copies for individual patients between “Pre” and “Post” timepoints. Purple solid lines: Non-Responders. Green dashed lines: Responders. Generally for the majority of subjects. (**C**) Boxplot displaying the calculated difference (Delta = Post − Pre) in the mtDNA copy number, stratified by response status. Individual data points are overlaid as black dots. Both Non-Responders (purple) and Responders (green) show a median decrease (negative delta) in copy number. (**D**) Statistical significance heatmap. A visualization of *p*-values derived from the analysis. Response Difference: Comparing Responders vs. Non-Responders yielded a non-significant *p*-value of 0.686 (Mann–Whitney U test). Time Effect: Comparing “Pre” vs. “Post” timepoints showed a statistically significant effect with a *p*-value of 0.023 (Wilcoxon signed-rank test, * *p*-value < 0.05).

DNA was extracted from the BMMCs of the HR-MDS patients’ bone marrow aspirates pre- and post-AZA therapy (Table 1). The extraction method followed preserves the nuclear as well as mitochondrial genomes within the same extracted DNA material. To obtain accurate photometric measurements for the DNA samples’ concentration, macromolecular DNA was mechanically sheared through a 21G syringe needle prior to use. PCR reactions were separately prepared for nuclear and mitochondrial sets of primers (Figure 4A) and run as duplicates or triplicates to confirm the reproducibility of results (Supplementary Figures S8 and S9).

Both Responders and Non-Responders to AZA treatment decreased their mtDNA copy number at the post-AZA time point (mtDNA median copy number pre-AZA 342.7 vs. post-AZA 246.1) (*p* = 0.023) (Figure 4B–D). The response to AZA was not determined by a differentiating criterion for the mtDNA copy number decline, as observed among the HR-MDS cohort studied. The reduction of mtDNA was observed in seven of the eight HR-MDS patients tested, with a median decline of 104 copies (28.8%). Patient NR3 showed a slight increase from 318.02 to 357.55 copies (Figure 4B). Recent evidence suggests the mtDNA copy number, a key component of mitochondrial content, is an important factor that reflects mtDNA replication and maintenance capability, directly coupled with mitochondrial biogenesis. Studies have discriminated between the proportion of mutated mtDNA copies, which effectively, but not sufficiently, designates the state of the disease and the absolute mtDNA copy number that also matters [25,52].

## 4. Discussion

As single-agent, AZA is still considered to be the first-line HMA treatment for HR-MDS, currently and over the past 15–20 years, but seems to have additional mechanisms of action outside of its role in DNA demethylation and reactivation of onco-suppressing genes. Among alternative AZA-associated mechanisms are the restoration of chromatin structure configuration [53], along with recombinational repair capability guided by long non-coding RNAs [54]. Another work refers to the beneficial modulation of autophagy in AZA-treated MDS patients [55].

Our results confirm the global nDNA demethylation AZA signatures (Figure 3A–C) that have been reported as the main and established function of AZA. As we have previously shown, Responders exhibit a significantly lower baseline global nDNA methylation compared to control samples, and further exhibit restricted demethylation capability post-AZA treatment. By contrast, Non-Responders show significant demethylation levels only after AZA treatment, reflecting a critical distinguishing factor between these groups [5]. The sample size of the present study (Table 1) limits the generalizability of results and our reported conclusions are cautious; however, we have performed a comprehensive, in-depth epigenetic study, employing highly accurate and valid methodologies, and producing scientifically meaningful results that emerge as potential biomarkers for discriminating Responders from Non-Responders to AZA therapy. Our results combine epigenetic and mitochondrial mechanisms of re-directing the neoplastic cells following treatment with AZA and are scientifically novel and sound remarks on the AZA-associated effects, confirming the general notification that AZA is primarily a disease-modifying and not a disease-eradicating agent. We also emphasize the biological rationale of clonal hematopoiesis manifested in MDS, suggesting that our results rather reflect changes in the HSPC compartment pre- and post-AZA treatment, although bulk bone marrow has been used in this study.

The discovery of RNA modifications has transformed our knowledge on biological functions, due to their direct or indirect implications in cell performance. However, normal levels of RNA-modified molecules in HSPCs, within the frame of hematologic pathologies, have not yet been precisely detected, primarily due to technical difficulties arising from their ephemeral nature and minimal amounts within cells. The evidence provided from our study, relying on the very sensitive and accurate analytical method of LC-MS/MS for their detection, shows that, among AZA Responders, Gm RNA modification is reduced. The m5C, m6A, m1A, and inosine RNA modifications also studied in the present work were either insignificantly altered or showed inconsistency (Figure 2). In the light of recent observations related to Gm functions—in terms that (i) “Gm readers” (binding proteins that preferentially bind to Gm-modified RNAs) have not been yet identified, (ii) Gm shows preference for weak binding to Piwi family proteins and Argonaute 2 (AGO2) [56,57], and (iii) the existence of an internal Gm site in the primary miRNA transcript let7a has been reported to inhibit the binding of microprocessor complex subunit DiGeorge syndrome critical region 8 (DGCR8) leading to the decreased let-7a maturation [58]—imply that Gm RNA modification favors interactions within the class of non-coding RNAs, suggesting its role in epigenome regulation. Furthermore, alteration of the other kinds of RNA-modifications studied (m6A, m5C, m1A) upon AZA therapy completion, even mildly, may facilitate suppression of malignant translational programs, such as tRNA charging efficiency (mainly in Responders), and eliminate the aberrant and high protein-synthesis upon which the neoplastic clones rely [46,47]. Conclusively, our study implicates Gm among the potential epigenetic factors influenced by AZA and provides an accurate method for its measurement.

In normal hematopoiesis, prostaglandins and related eicosanoids regulate HSPCs’ quiescence, influence myeloid differentiation, and shape the bone marrow niche, including stromal and immune cells. Short-term exposure of HSPCs to prostaglandin E2 (PGE2) boosts homing, survival, and proliferation, increasing their repopulating ability [59]. In the HR-MDS Responders of the present study, the restoration of a controlled inflammatory responsiveness regulated by miRNA families (Figure 1) might represent a prerequisite for effective differentiation. Salutary phenotypic effects are the improved myeloid maturation implying the underlying re-induction of responsiveness to differentiation cues or the re-engagement of innate productive immune signaling. This aligns well with the concept that AZA response requires immune reprogramming, not just impaired immune silencing. Additionally, among HR-MDS Responders, decreased miRNA family members that suppress glycolysis offer metabolic flexibility, improvement in pyruvate metabolism, and glycolytic pathway, thus representing a strong signal for the cell-state transition to an active differentiating state, approaching a normal HSPC life cycle [60]. Leukemic stem cells (LSCs) have been reported to show rigid metabolic wiring, often with specific, fixed metabolic dependencies. LSCs in de novo AML heavily rely on amino acid breakdown forwarded to OXHPOS to produce energy, a weakness targeted by the combination of AZA and venetoclax therapy [61,62], while other published work has reported elevated levels of fatty acid oxidation within the mitochondria of some LSC subpopulations [63]. Glycolysis awakening, obtained in the present study among the Responders’ group of HR-MDS patients, may redirect HSPCs metabolism to a state less supported by OXHPOS, and may also engage epigenetic remodeling by enhancement of one-carbon metabolism. Glycolytic reprogramming promotes changes in key metabolites, such as acetyl-Coenzyme A (CoA), S-adenosylmethionine (SAM), nicotinamide adenine dinucleotide (NAD^+^/NADH), and α-ketoglutarate (α-KG), among others. These factors function as direct cofactors or substrates of epigenetic-related enzymes; histone acetyltransferases, DNA and histone methyltransferases, sirtuins, ten-eleven translocation 1–3 enzymes (TET1–3), and others, affecting enzyme activity [64,65] and assisting AZA response [66]. Metabolic pathway changes related to the AZA treatment in Responders detected in our study are likely to contribute both to potential alterations of aberrant chromatin modifications and to gene expression programs that control stemness, self-renewal, and HSPC differentiation, with consequent favorable outcomes at the clinical level.

Moreover, AZA positively affects the ribosomal protein function in the HR-MDS Responders of the present study, and this could mainly be attributed to the reversed epigenetic silencing and normalization of post-transcriptional networks, controlling ribosome biogenesis and translation. Although a previously published work has demonstrated that AZA treatment failure is associated with the upregulation of ribosomal genes and pathways related to ribosomal stress [67], our results suggest a ribosomal function recovery in HR-MDS Responders,. Our results are also in line with studies reporting significant improvement of rRNA re-expression and nucleolar function restoration in CD34+ cells from MDS patients after treatment with AZA, which can be attributed to the decrease of rDNA promoter methylation [68].

Another hallmark of MDS is that dysplastic HSPCs are in high metabolic stress and rely on survival programs, executing “response to starvation” pathways when cells sense nutrient or energy insufficiency. A deep transcriptomic analysis conducted in MDS revealed that a number of molecular functions, such as cellular respiration and apoptosis, were relatively activated only in Responders to therapy [69]. In our study, AZA effects indicated an improved perceived nutrient availability guided by reprogrammed metabolic pathways that were influenced by differently expressed miRNA networks. Furthermore, upregulated sets of miRNAs responsible for directing the “response to starvation” program were observed post-AZA treatment, suggesting its suppression in HR-MDS Responders. This epigenetic redirection may also favor ribosome biogenesis and translation over stress conservation (Figure 1). Overall, AZA response seems to confer a survival benefit, not just by acting as a hypomethylating agent, but by offering several epigenetic functional restatements.

The absolute mtDNA copy number increase, which has been observed in various hematological malignancies, is puzzling. The results from pediatric patients with acute lymphoblastic leukemia (ALL) have shown significantly higher copy numbers of mtDNA in bone marrow at diagnosis, when compared with controls. Monitoring the same cohort post-standard chemotherapy treatment, it was found that the mtDNA copy number decreased. The elevated mtDNA copy number at the time of initial MDS diagnosis has been associated with inferior survival and has been proposed as a potential marker of low therapeutic efficacy [70]. An elevated mtDNA copy number also occurs in pediatric AML and has been considered to be an independent predictor of poor event-free and overall survival [71]. In adult AML patients treated with standard intensive chemotherapy, a high mtDNA content has been associated with chemoresistance and predicted worse relapse-free survival and resistance to cytarabine-based therapy [72]. However, increased mtDNA copy numbers could efficiently overcome the bioenergetic defects induced by some (often detected) mtDNA mutations and could represent a compensatory mechanism aiming to sustain OXHPOS activity, thus delaying the onset of disease manifestations, which has been documented in mice [73]. In humans, high mtDNA copy numbers in the blood have been associated with an increased risk of developing lymphomas [74].

In consistency with these reports, a significant decrease in the mtDNA copy number in the HR-MDS cohort was detected post-AZA treatment (Figure 4). Our results linked AZA treatment with mitochondrial depletion and potential development of partial OXPHOS independency, and also designated a synergistic mechanism of a favorable response to AZA, guiding HSPCs to switch toward a glycolytic metabolism and ribosomal rebound. Simultaneously with the mtDNA significant decrease, elevated HSPCs’ survival could be supported, preventing at the same time malignant progression in AZA Responders. Within this frame, Non-Responders, even though they achieve a decrease in mtDNA copy number, cannot successfully control the metabolic shift, and therefore remain with a critical disadvantage to obtain benefit from AZA. Taken together, the link between the metabolic restoration of proliferative programs (glycolysis, translation, ribosome biogenesis) with active suppression of mitochondrial (OXHPOS) dependence and the reduction of mtDNA copy number observed in HR-MDS Responders, suggests that HSPCs’ repair, recover, and direct cells to a survival mode, fitting to a therapy-induced, growth-arrested, but biosynthetically active cell state. However, despite meaningful clinical improvements from AZA therapy, sustained responses are uncommon and more often are short-lived.

## 5. Conclusions

Results of the present study apply to a limited number of HR-MDS patients and further confirmation from a statistical sizeable patient cohort is necessary. To summarize, AZA effects in the epigenome have highlighted the reduction of Gm RNA modification in HR-MDS Responders and the positive regulation of specific miRNA families, leading to the potential restoration of several critical cell pathways: prostaglandin synthesis and regulation, ribosomal protein synthesis and function, as well as glycolysis. Furthermore, response to starvation was suppressed, offering overall unique benefits to the MDS clinical phenotype, and defined as response to AZA therapy. At the level of mitochondrial biogenesis, AZA significantly reduced the copies of mtDNA in both Responders and Non-Responders among the studied HR-MDS cohort, an AZA signature that merits further research and decoding for its role in the clinical outcome.

## Figures and Tables

**Table 1:** Clinical data of the HR-MDS patients’ cohort.

Sample	Sex	Age *	MDS Type	Charlson’s Comorbidity Index	Karyotype	Transfusion Dependency	BM Blasts	Mutations	IPSS-R/IPSS-M *	Type of Response	AML Progress During Treatment *
R1-PRE	F	67	MDS-MLD	3	45, XX, −7 [6/20]	No	3%	*ASXL1*, *EZH2*, *SETBP1*, *STAG2*	6.5 /1.42	mCR+HI	NO
R1-POST			MDS-MLD		46, XX [20]	No	1%				
R2-PRE	M	70	MDS-EB1	4	47, XY, +8 [2/20]	Yes	16%	N/A	6.0/1.34	CR	NO
R2-POST			MDS-MLD		46, XY [20]	No	3.5%				
R3-PRE	M	60	MDS-EB1	5	46, XY [20]	Yes	6.6%	*RUNX1*	5.0/1.09	mCR+HI	NO
R3-POST			MDS-MLD		Ν/A	No	4.5%				
R4-PRE	M	71	MDS-EB2	6	46, XY [20]	No	11%	N/A	4.5/0.58	mCR+HI	NO
R4-POST			MDS-EB1		46, XY [20]	No	6%				
NR1-PRE	F	76	MDS-EB1	3	46, ΧΧ [20]	No	5.5%	*ASXL1*, *TET2*	5.0/−0.12	NR/SD	NO
NR1-POST			MDS-MLD		46, ΧΧ [20]	No	4.5%				
NR2-PRE	F	58	MDS-MLD	3	46, XX [20]	Yes	1.5%	N/A	2.5/−0.47	NR/SD	NO
NR2-POST			MDS-MLD		Ν/A	Yes	3.3%				
NR3-PRE	M	85	MDS-EB2	5	46, XY [20]	No	12%	N/A	6.0/1.52	NR	NO
NR3-POST			MDS-EB1		N/A	No	11%	*RUNX1*, *U2AF1*, *JAK2*			
NR4-PRE	M	69	MDS-EB1	5	46, XY [20]	Yes	7%	*TET2*	5.5/0.28	NR	YES (6)
NR4-POST			MDS-EB2/AML		N/A	Yes	25%	*RUNX1*, *ASXL1*, *TET2*, *U2AF1*			

* Age in years, Response duration/Time to AML/OS in months, IPSS-R/IPSS-M: Revised/Molecular International Prognostic Scoring System tools for risk stratification of MDS.

## Data Availability

The NGS data have been deposited in NCBI’s Gene Expression Omnibus and are accessible through GEO Series accession number GSE315656 “https://www.ncbi.nlm.nih.gov/geo/query/acc.cgi?acc=GSE315656” (accessed on 5 January 2026). Digital PCR and LC-MS/MS data of the present study can be provided upon request. All custom codes, pre-processing automation frameworks, and statistical calculation blocks are publicly hosted on GitHub (https://github.com/irenenter-maker/MDS-miRNA-AZA-pipeline (accessed on 5 January 2026)).

## Supplementary Materials

The following supporting information can be downloaded at: https://www.mdpi.com/article/10.3390/cancers18142305/s1, Table S1. Supplementary sequencing statistics and quality control metrics across samples. Table S2. Top 20 miRNAs ranked by unadjusted p-value for the response × time interaction. Table S3. LOD and LOQ for each RNA modification (LC-MS/MS). Table S4. Intra-day precision (%RSD) per RNA modification. Table S5. Intra-day precision (repeatability) for RNA modification measurements (duplicate injections per sample). Figure S1. Volcano plot of miRNA differential expression for the response × time interaction. Figure S2. Signaling Pathways Modulated by AZA Response (miRWalk). Figure S3. Significant Enriched GO Terms (miRWalk). Figure S4. Enrichment of miRNA Target Genes (miRTarBase). Figure S5. Biological Processes Enriched in AZA Response (miRPathDB). Figure S6. Network Analysis of miRNA Clusters and Targets. Figure S7. Network Analysis of miRNA Families and Targets. Figure S8. Digital PCR (dPCR) scatter plots for target genes GAPDH and MT-CYB of HR-MDS DNA sample pre- and post-AZA treatment. Figure S9. Assessment of the repeatability and linearity of the digital PCR (dPCR) assay.

## Abbreviations

The following abbreviations are used in this manuscript:

ACN-HCOOH	Acidified acetonitrile
AGO2	Argonaute RISC catalytic component 2
A → I	Adenosine-to-inosine editing
α-KG	α-ketoglutarate
ALL	Acute lymphoblastic leukemia
AML	Acute myeloid leukemia
AZA	Azacitidine
BMMCs	Bone marrow mononuclear cells
CoA	Acetyl-coenzyme A
CLEC12B	C-Type lectin domain family 12 member B
CMML	Chronic myelomonocytic leukemia
Co-A	Coenzyme A
CTCF	CCCTC-binding factor
DEA	Differential expression analysis
dPCR	Digital polymerase chain reaction
EPO	Erythropoietin
ESI	Electrospray ionization system
FASTQ	Raw sequencing data
FBXL5	F-box and leucine-rich repeat protein 5
GAPDH	Glyceraldehyde-3-phosphate dehydrogenase
GATA2	Globin transcription factor 2
Gm	2′-O-Methyl-guanosine RNA modification
GO	Gene ontology
GSEA	Gene set enrichment analysis
HMAs	Hypomethylating agents
HOXA	Homeobox A cluster
HOXB	Homeobox B cluster
HR-MDS	Higher-risk MDS
HSPCs	Hematopoietic stem and progenitor cells
IWG	International Working Group
KCNB1	Potassium voltage-gated channel subfamily B member 1
LC	Liquid chromatography
LC-MS/MS	Liquid chromatography combined with mass spectrometry
lncRNAs	Long non-coding ribonucleic acids
LSCs	Leukemic stem cells
m1A	N1-methyladenosine RNA modification
m6A	N6-methyladenosine RNA modification
m5C	5-methylcytosine RNA modification
MDS	Myelodysplastic syndromes
MDS-EB	Myelodysplastic syndromes with an excess of marrow blasts
MDS-MLD	Myelodysplastic syndromes with multilineage dysplasia
METTL14	Methyltransferase 14, m6A methyltransferase
miRNA	MicroRNA
MRM	Multiple reaction monitoring
mtDNA	Mitochondrial DNA
NAD^+^/NADH	Nicotinamide adenine dinucleotide
nDNA	Nuclear deoxyribonucleic acid
NES	Normalized enrichment score
NGS	Next generation sequencing
OXPHOS	Oxidative phosphorylation
PCR	Polymerase chain reaction
PGE2	Prostaglandin E2
RIS	Integrity Scores
SAM	S-Adenosyl methionine
SNAI1	Snail family transcriptional repressor 1
STAG2	STAG2 cohesin complex component
SF3B1	Splicing factor 3b subunit 1
TET1-3	Ten-eleven translocation 1–3 enzymes
TRIM37	Tripartite motif containing 37
U2AF1	U2 small nuclear RNA auxiliary factor 1
UMIs	Unique molecular identifiers

## References

[1] Kuendgen A., Müller-Thomas C., Lauseker M., Haferlach T., Urbaniak P., Schroeder T., Brings C., Wulfert M., Meggendorfer M., Hildebrandt B. (2018). Efficacy of azacitidine is independent of molecular and clinical characteristics—An analysis of 128 patients with myelodysplastic syndromes or acute myeloid leukemia and a review of the literature. Oncotarget.

[2] Hansen A.S. (2020). CTCF as a boundary factor for cohesin-mediated loop extrusion: Evidence for a multi-step mechanism. Nucleus.

[3] Thoms J.A.I., Yan F., Hampton H.R., Davidson S., Joshi S., Saw J., Sarowar C.H., Lim X.Y., Nunez A.C., Kakadia P.M. (2025). Clinical response to azacitidine in MDS is associated with distinct DNA methylation changes in HSPCs. Nat. Commun..

[4] Tran H.T.T., Kim H.N., Lee I.-K., Kim Y.-K., Ahn J.-S., Yang D.-H., Lee J.-J., Kim H.-J. (2011). DNA Methylation Changes Following 5-azacitidine Treatment in Patients with Myelodysplastic Syndrome. J. Korean Med. Sci..

[5] Nikolopoulos T., Bochalis E., Chatzilygeroudi T., Chondrou V., Dereki I., Athanasopoulou K., Zafeiropoulos J., Bourikas K., Patrinos G.P., Symeonidis A. (2025). Integrating advanced analytical methods to assess epigenetic marks affecting response to hypomethylating agents in higher risk myelodysplastic syndrome. Mol. Med..

[6] Pellagatti A., Boultwood J. (2021). SF3B1 mutant myelodysplastic syndrome: Recent advances. Adv. Biol. Regul..

[7] Li B., Liu J., Jia Y., Wang J., Xu Z., Qin T., Shi Z., Song Z., Peng S., Huang H. (2018). Clinical features and biological implications of different *U2AF1* mutation types in myelodysplastic syndromes. Genes Chromosomes Cancer.

[8] Lev Maor G., Yearim A., Ast G. (2015). The alternative role of DNA methylation in splicing regulation. Trends Genet..

[9] Shayevitch R., Askayo D., Keydar I., Ast G. (2018). The importance of DNA methylation of exons on alternative splicing. RNA.

[10] Pellagatti A., Boultwood J. (2023). Splicing factor mutations in the myelodysplastic syndromes: Role of key aberrantly spliced genes in disease pathophysiology and treatment. Adv. Biol. Regul..

[11] Wang X., Zhao B.S., Roundtree I.A., Lu Z., Han D., Ma H., Weng X., Chen K., Shi H., He C. (2015). N6-methyladenosine Modulates Messenger RNA Translation Efficiency. Cell.

[12] Pilala K.-M., Panoutsopoulou K., Papadimitriou M.-A., Soureas K., Scorilas A., Avgeris M. (2025). Exploring the methyl-verse: Dynamic interplay of epigenome and m6A epitranscriptome. Mol. Ther..

[13] Symeonidis A., Chroni A., Dereki I., Chartoumpekis D., Sgourou A. (2025). Intracellular Mis-Localization of Modified RNA Molecules and Non-Coding RNAs: Facts from Hematologic Malignancies. Curr. Issues Mol. Biol..

[14] Jiang L., Zhang Y., Qian J., Zhou X., Ma L., Zhu S., Wang L., Wang W., Yang W., Luo Y. (2024). The m6A methyltransferase METTL14 promotes cell proliferation via SETBP1-mediated activation of PI3K-AKT signaling pathway in myelodysplastic neoplasms. Leukemia.

[15] Liu F., Wang M., Gao S., Song G., Liu M., Li Y., Sun P., Lai W., Wang H., Yang Y.-G. (2025). RNA m5C methylation mediated by Ybx1 ensures hematopoietic stem and progenitor cell expansion. Cell Rep..

[16] Zuo H., Wu A., Wang M., Hong L., Wang H. (2024). tRNA m1A modification regulate HSC maintenance and self-renewal via mTORC1 signaling. Nat. Commun..

[17] Wang Q., Khillan J., Gadue P., Nishikura K. (2000). Requirement of the RNA Editing Deaminase ADAR1 Gene for Embryonic Erythropoiesis. Science.

[18] Chen X., Yuan Y., Zhou F., Li L., Pu J., Jiang X. (2024). RNA modification in normal hematopoiesis and hematologic malignancies. MedComm.

[19] Krejcik Z., Belickova M., Hrustincova A., Votavova H., Jonasova A., Cermak J., Dyr J.E., Merkerova M.D. (2018). MicroRNA profiles as predictive markers of response to azacitidine therapy in myelodysplastic syndromes and acute myeloid leukemia. Cancer Biomark..

[20] Mongiorgi S., De Stefano A., Ratti S., Indio V., Astolfi A., Casalin I., Pellagatti A., Paolini S., Parisi S., Cavo M. (2023). A miRNA screening identifies miR-192-5p as associated with response to azacitidine and lenalidomide therapy in myelodysplastic syndromes. Clin. Epigenet..

[21] Shin M.G., Kajigaya S., Levin B.C., Young N.S. (2003). Mitochondrial DNA mutations in patients with myelodysplastic syndromes. Blood.

[22] Wulfert M., Küpper A.C., Tapprich C., Bottomley S.S., Bowen D., Germing U., Haas R., Gattermann N. (2008). Analysis of mitochondrial DNA in 104 patients with myelodysplastic syndromes. Exp. Hematol..

[23] Kornicka K., Marycz K., Marędziak M., Tomaszewski K.A., Nicpoń J. (2017). The effects of the DNA methyltranfserases inhibitor 5-Azacitidine on ageing, oxidative stress andDNA methylation of adipose derived stem cells. J. Cell. Mol. Med..

[24] Schildgen V., Wulfert M., Gattermann N. (2011). Impaired mitochondrial gene transcription in myelodysplastic syndromes and acute myeloid leukemia with myelodysplasia-related changes. Exp. Hematol..

[25] Filograna R., Mennuni M., Alsina D., Larsson N. (2021). Mitochondrial DNA copy number in human disease: The more the better?. FEBS Lett..

[26] Gulei D., Moisoiu V., Kegyes D., Drula R., Iluta S., Tigu A.B., Nistor M., Jitaru C., Bancos A., Rotariu P. (2024). RNA methylation sequencing shows different gene expression signatures for response to azacytidine therapy in high-grade myelodysplastic syndromes. J. Cell. Mol. Med..

[27] Komrokji R.S., Al Ali N.H., Sallman D., Padron E., DeZern A.E., Barnard J., Roboz G.J., Garcia-Manero G., List A., Steensma D.P. (2021). Validation of International Working Group response criteria in higher-risk myelodysplastic syndromes: A report on behalf of the MDS Clinical Research Consortium. Cancer Med..

[28] Platzbecker U., Fenaux P., Adès L., Giagounidis A., Santini V., van de Loosdrecht A.A., Bowen D., de Witte T., Garcia-Manero G., Hellström-Lindberg E. (2019). Proposals for revised IWG 2018 hematological response criteria in patients with MDS included in clinical trials. Blood.

[29] Spella M., Bochalis E., Athanasopoulou K., Chroni A., Dereki I., Ntaliarda G., Makariti I., Psarias G., Constantinou C., Chondrou V. (2024). Crosstalk between non-coding RNAs and transcription factor LRF in non-small cell lung cancer. Noncoding RNA Res..

[30] Smith T., Heger A., Sudbery I. (2017). UMI-tools: Modeling sequencing errors in Unique Molecular Identifiers to improve quantification accuracy. Genome Res..

[31] Martin M. (2011). Cutadapt removes adapter sequences from high-throughput sequencing reads. EMBnet J..

[32] Kozomara A., Birgaoanu M., Griffiths-Jones S. (2019). miRBase: From microRNA sequences to function. Nucleic Acids Res..

[33] Li H., Durbin R. (2009). Fast and accurate short read alignment with Burrows–Wheeler transform. Bioinformatics.

[34] Li H., Handsaker B., Wysoker A., Fennell T., Ruan J., Homer N., Marth G., Abecasis G., Durbin R. (2009). The Sequence Alignment/Map format and SAMtools. Bioinformatics.

[35] Love M.I., Huber W., Anders S. (2014). Moderated estimation of fold change and dispersion for RNA-seq data with DESeq2. Genome Biol..

[36] Korotkevich G., Sukhov V., Budin N., Shpak B., Artyomov M.N., Sergushichev A. (2016). Fast gene set enrichment analysis. bioRxiv.

[37] Kern F., Fehlmann T., Solomon J., Schwed L., Grammes N., Backes C., Van Keuren-Jensen K., Craig D.W., Meese E., Keller A. (2020). miEAA 2.0: Integrating multi-species microRNA enrichment analysis and workflow management systems. Nucleic Acids Res..

[38] Lin X., Zhang Q., Qin Y., Zhong Q., Lv D., Wu X., Fu P., Lin H. (2022). Potential Misidentification of Natural Isomers and Mass-Analogs of Modified Nucleosides by Liquid Chromatography–Triple Quadrupole Mass Spectrometry. Genes.

[39] Ogawa A., Wei F.-Y. (2021). Protocol for preparation and measurement of intracellular and extracellular modified RNA using liquid chromatography-mass spectrometry. STAR Protoc..

[40] Kozhukhar N., Fant A., Alexeyev M.F. (2021). Quantification of mtDNA content in cultured cells by direct droplet digital PCR. Mitochondrion.

[41] Sticht C., De La Torre C., Parveen A., Gretz N. (2018). miRWalk: An online resource for prediction of microRNA binding sites. PLoS ONE.

[42] Kehl T., Kern F., Backes C., Fehlmann T., Stöckel D., Meese E., Lenhof H.-P., Keller A. (2020). miRPathDB 2.0: A novel release of the miRNA Pathway Dictionary Database. Nucleic Acids Res..

[43] Patil D.P., Pickering B.F., Jaffrey S.R. (2018). Reading m6A in the Transcriptome: M6A-Binding Proteins. Trends Cell Biol..

[44] Guan Z., Li W., He Y., Guo W. (2025). RNA m5C methylation in cancer: Mechanisms and biological impact. Oncogenesis.

[45] Dominissini D., Nachtergaele S., Moshitch-Moshkovitz S., Peer E., Kol N., Ben-Haim M.S., Dai Q., Di Segni A., Salmon-Divon M., Clark W.C. (2016). The dynamic N1-methyladenosine methylome in eukaryotic messenger RNA. Nature.

[46] Oerum S., Dégut C., Barraud P., Tisné C. (2017). m1A Post-Transcriptional Modification in tRNAs. Biomolecules.

[47] Shima H., Igarashi K. (2020). N 1-methyladenosine (m1A) RNA modification: The key to ribosome control. J. Biochem..

[48] Marchand V., Pichot F., Thüring K., Ayadi L., Freund I., Dalpke A., Helm M., Motorin Y. (2017). Next-Generation Sequencing-Based RiboMethSeq Protocol for Analysis of tRNA 2′-O-Methylation. Biomolecules.

[49] Levanon E.Y., Eisenberg E., Yelin R., Nemzer S., Hallegger M., Shemesh R., Fligelman Z.Y., Shoshan A., Pollock S.R., Sztybel D. (2004). Systematic identification of abundant A-to-I editing sites in the human transcriptome. Nat. Biotechnol..

[50] Liu Z., Tian J., Peng F., Wang J. (2022). Hypermethylation of mitochondrial DNA facilitates bone metastasis of renal cell carcinoma. J. Cancer.

[51] Shao Z., Han Y., Zhou D. (2023). Optimized bisulfite sequencing analysis reveals the lack of 5-methylcytosine in mammalian mitochondrial DNA. BMC Genom..

[52] Moraes C.T. (2001). What regulates mitochondrial DNA copy number in animal cells?. Trends Genet..

[53] Xu J., Song F., Lyu H., Kobayashi M., Zhang B., Zhao Z., Hou Y., Wang X., Luan Y., Jia B. (2022). Subtype-specific 3D genome alteration in acute myeloid leukaemia. Nature.

[54] Merkerova M.D., Klema J., Kundratd D., Sziksazi K., Krejcik Z., Hrustincova A., Trsova I., Le A.V., Cermak J., Jonasova A. (2022). Noncoding RNAs and Their Response Predictive Value in Azacitidine-treated Patients With Myelodysplastic Syndrome and Acute Myeloid Leukemia With Myelodysplasia-related Changes. Cancer Genom. Proteom..

[55] Tsekoura G., Agathangelidis A., Kontandreopoulou C.-N., Fasouli E.S., Katsantoni E., Pliaka V., Alexopoulos L., Katana E., Papaioannou M., Taktikou G. (2025). Restoration of Autophagy and Apoptosis in Myelodysplastic Syndromes: The Effect of Azacitidine in Disease Pathogenesis. Curr. Issues Mol. Biol..

[56] Tian Y., Simanshu D.K., Ma J.-B., Patel D.J. (2011). Structural basis for piRNA 2′-O-methylated 3′-end recognition by Piwi PAZ (Piwi/Argonaute/Zwille) domains. Proc. Natl. Acad. Sci. USA.

[57] Liang H., Jiao Z., Rong W., Qu S., Liao Z., Sun X., Wei Y., Zhao Q., Wang J., Liu Y. (2020). 3′-Terminal 2′-O-methylation of lung cancer miR-21-5p enhances its stability and association with Argonaute 2. Nucleic Acids Res..

[58] Bian Z., Xu C., Xie Y., Wang X., Chen Y., Mao S., Wu Q., Zhu J., Huang N., Zhang Y. (2023). SNORD11B-mediated 2′-O-methylation of primary let-7a in colorectal carcinogenesis. Oncogene.

[59] Hoggatt J., Singh P., Sampath J., Pelus L.M. (2009). Prostaglandin E2 enhances hematopoietic stem cell homing, survival, and proliferation. Blood.

[60] Lagadinou E.D., Sach A., Callahan K., Rossi R.M., Neering S.J., Minhajuddin M., Ashton J.M., Pei S., Grose V., O’Dwyer K.M. (2013). BCL-2 Inhibition Targets Oxidative Phosphorylation and Selectively Eradicates Quiescent Human Leukemia Stem Cells. Cell Stem Cell.

[61] Jones C.L., Stevens B.M., D’Alessandro A., Reisz J.A., Culp-Hill R., Nemkov T., Pei S., Khan N., Adane B., Ye H. (2018). Inhibition of Amino Acid Metabolism Selectively Targets Human Leukemia Stem Cells. Cancer Cell.

[62] Chatzilygeroudi T., Karantanos T., Pappa V. (2025). Unraveling Venetoclax Resistance: Navigating the Future of HMA/Venetoclax-Refractory AML in the Molecular Era. Cancers.

[63] Ye H., Adane B., Khan N., Sullivan T., Minhajuddin M., Gasparetto M., Stevens B., Pei S., Balys M., Ashton J.M. (2016). Leukemic Stem Cells Evade Chemotherapy by Metabolic Adaptation to an Adipose Tissue Niche. Cell Stem Cell.

[64] Agrawal-Singh S., Bagri J., Sakakini N., Huntly B.J.P. (2023). A guide to epigenetics in leukaemia stem cells. Mol. Oncol..

[65] Ryall J.G., Cliff T., Dalton S., Sartorelli V. (2015). Metabolic Reprogramming of Stem Cell Epigenetics. Cell Stem Cell.

[66] Bejar R., Lord A., Stevenson K., Bar-Natan M., Pérez-Ladaga A., Zaneveld J., Wang H., Caughey B., Stojanov P., Getz G. (2014). TET2 mutations predict response to hypomethylating agents in myelodysplastic syndrome patients. Blood.

[67] Monika Belickova M., Merkerova M.D., Votavova H., Valka J., Vesela J., Pejsova B., Hajkova H., Klema J., Cermak J., Jonasova A. (2016). Up-regulation of ribosomal genes is associated with a poor response to azacitidine in myelodysplasia and related neoplasms. Int. J. Hematol..

[68] Raval A., Sridhar K.J., Patel S., Turnbull B.B., Greenberg P.L., Mitchell B.S. (2012). Reduced rRNA expression and increased rDNA promoter methylation in CD34+ cells of patients with myelodysplastic syndromes. Blood.

[69] Kim K., Park S., Choi H., Kim H.J., Kwon Y.-R., Ryu D., Kim M., Kim T.-M., Kim Y.-J. (2020). Gene expression signatures associated with sensitivity to azacitidine in myelodysplastic syndromes. Sci. Rep..

[70] Jain A., Bakhshi S., Thakkar H., Gerards M., Singh A. (2018). Elevated mitochondrial DNA copy numbers in pediatric acute lymphoblastic leukemia: A potential biomarker for predicting inferior survival. Pediatr. Blood Cancer.

[71] Chaudhary S., Ganguly S., Palanichamy J.K., Singh A., Bakhshi R., Jain A., Chopra A., Bakhshi S. (2021). PGC1A driven enhanced mitochondrial DNA copy number predicts outcome in pediatric acute myeloid leukemia. Mitochondrion.

[72] Pereira-Martins D.A., Weinhäuser I., Griessinger E., Coelho-Silva J.L., Silveira D.R., Sternadt D., Erdem A., Duarte B.K.L., Chatzikyriakou P., Quek L. (2025). High mtDNA content identifies oxidative phosphorylation-driven acute myeloid leukemias and represents a therapeutic vulnerability. Signal Transduct. Target. Ther..

[73] Filograna R., Koolmeister C., Upadhyay M., Pajak A., Clemente P., Wibom R., Simard M.L., Wredenberg A., Freyer C., Stewart J.B. (2019). Modulation of mtDNA copy number ameliorates the pathological consequences of a heteroplasmic mtDNA mutation in the mouse. Sci. Adv..

[74] Mi J., Tian G., Liu S., Li X., Ni T., Zhang L., Wang B. (2015). The Relationship Between Altered Mitochondrial DNA Copy Number And Cancer Risk: A Meta-Analysis. Sci. Rep..

