# Supplementary material for: RNA and Mitochondrial Reprogramming Associated with Azacytidine Treatment in Higher-Risk Myelodysplastic Syndromes: A Pilot Study
Source: Cancers (Basel). 2026 Jul 17;18(14):2305. doi: 10.3390/cancers18142305 (PMC13406402; doi:10.3390/cancers18142305)
Supplement: Supplementary file 1 [file cancers-18-02305-s001.zip › cancers-4415636-supplementary.pdf]

Supplementary Table S1. Supplementary sequencing statistics and quality control metrics across samples.
Comprehensive overview of processing throughput, sequencing depth, and alignment success for individual pre- and post-treatment samples. Columns report raw sequencing depth (Raw Reads), reads passing unique molecular identifier (UMI) extraction, and post-trimming yield (Trimmed Reads). The percentage of reads within the characteristic 18-25 nt mature miRNA length range is provided alongside total mapped reads and corresponding mapping percentages. High-confidence alignments are indicated by MAPQ ≥ 30 counts, while final Unique Molecules reflect the absolute biological signal remaining after de-duplication via umi_tools. PCR duplication rates are reported as Duplicates (%).

Supplementary Sequencing Statistics and Quality Control Metrics

Sample ID	Treatment	Raw Reads	UMI Extracted	Trimmed Reads	18-25nt (%)	Mapped Reads	Mapped (%)	MAPQ ≥ 30	MAPQ ≥ 30 (%)	Unique Molecules	Duplicates (%)
NR1	Pre	1367625	1314908	852667	8801	535796	6284	404234	4741	468647	12.53
NR1	Post	1405508	1339625	875800	8315	486761	5558	366836	4189	425510	12.58
NR2	Post	794219	718874	533507	9235	360421	6756	270906	5078	309492	14.13
NR2	Pre	580951	526091	300032	8599	172268	5742	124749	4158	159417	7.46
NR3	Pre	880087	803233	463636	9074	312868	6748	216680	4673	267626	14.46
NR3	Post	1000916	932620	614269	5429	237473	3866	167649	2729	201574	15.12
NR4	Pre	893074	813473	535387	9605	381054	7117	318917	5957	339089	11.01
NR4	Post	833177	763602	481997	9156	308677	6404	220038	4565	286117	7.31
R1	Post	1329284	1294035	768406	8953	480424	6252	361962	4711	409333	14.80
R1	Pre	1266845	1227945	923431	8906	573813	6214	453774	4914	503190	12.31
R2	Post	1252935	1205299	745368	8561	368697	4947	305098	4093	312094	15.35
R2	Pre	1288536	1273797	932294	6941	378403	4059	318309	3414	326680	13.67
R3	Pre	983183	775902	619612	8019	331217	5346	249347	4024	302679	8.62
R3	Post	1045935	1009228	780960	8944	411606	5271	324766	4159	375616	8.74

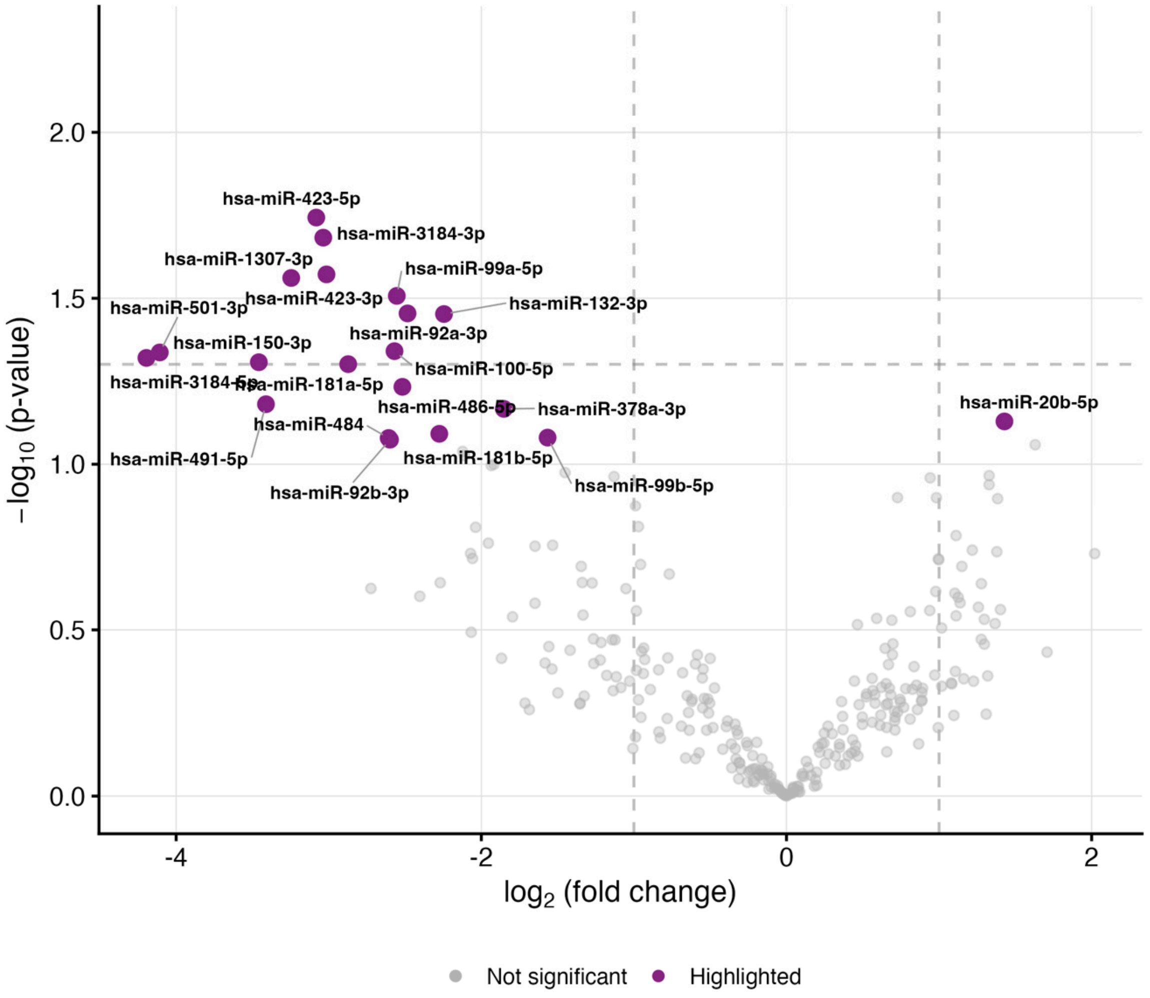

Figure S1. Volcano plot of miRNA differential expression for the response × time interaction.

The $\log_2$(fold change) is plotted against the $-\log_{10}$(unadjusted p-value) for all quantified mature miRNAs evaluated via DESeq2. Dashed vertical lines represent absolute $\log_2$(fold change) thresholds of 1, and the dashed horizontal line indicates an unadjusted p-value threshold of 0.05 ($-\log_{10}(p) \approx 1.30$). Purple points denote the top 20 ranked miRNAs prioritized by nominal, unadjusted significance (p-value). No individual miRNAs achieved strict statistical significance after Benjamini-Hochberg false discovery rate correction (adjusted p-value<0.05), validating the utilization of competitive Gene Set Enrichment Analysis (GSEA) to evaluate biological pathways.

Supplementary Table S2. Top 20 miRNAs ranked by unadjusted p-value for the response × time interaction.
Differential expression metrics generated by DESeq2 for the top 20 candidate miRNAs prioritized by nominal statistical significance (unadjusted p-value). Columns define the base mean of normalized counts across all samples, the Wald test statistic, log_2(fold change) variations of temporal change between Responders and Non-Responders, the nominal unadjusted p-value, and the Benjamini-Hochberg adjusted p-value.

Top 20 miRNAs ranked by unadjusted p-value for the response × time interaction.

miRNA	Base mean	Wald statistic	log2(Fold change)	p-value	Adjusted p-value
hsa-miR-423-5p	185.425377	-2.364305	-3.081790	0.0180639	0.9599361
hsa-miR-3184-3p	182.874889	-2.312183	-3.035937	0.0207676	0.9599361
hsa-miR-423-3p	115.634391	-2.214482	-3.014902	0.0267957	0.9599361
hsa-miR-1307-3p	23.700663	-2.204777	-3.245921	0.0274697	0.9599361
hsa-miR-99a-5p	43.856087	-2.155756	-2.554088	0.0311027	0.9599361
hsa-miR-92a-3p	2847.301262	-2.106868	-2.483705	0.0351290	0.9599361
hsa-miR-132-3p	13.879823	-2.105185	-2.244793	0.0352752	0.9599361
hsa-miR-100-5p	36.040454	-1.998688	-2.569056	0.0456422	0.9599361
hsa-miR-501-3p	6.587949	-1.995001	-4.107383	0.0460428	0.9599361
hsa-miR-3184-5p	10.240398	-1.978903	-4.195087	0.0478269	0.9599361
hsa-miR-150-3p	7.815989	-1.966131	-3.458360	0.0492835	0.9599361
hsa-miR-181a-5p	483.805387	-1.960434	-2.872640	0.0499451	0.9599361
hsa-miR-486-5p	2117.955825	-1.892263	-2.517361	0.0584559	0.9599361
hsa-miR-491-5p	4.100176	-1.838781	-3.411432	0.0659473	0.9599361
hsa-miR-378a-3p	140.314440	-1.824204	-1.852300	0.0681213	0.9599361
hsa-miR-20b-5p	278.171375	1.784705	1.428177	0.0743091	0.9599361
hsa-miR-181b-5p	63.689865	-1.745026	-2.275524	0.0809803	0.9599361
hsa-miR-99b-5p	83.370470	-1.732736	-1.565870	0.0831426	0.9599361
hsa-miR-484	84.023609	-1.731515	-2.607429	0.0833599	0.9599361
hsa-miR-92b-3p	15.073955	-1.725972	-2.598652	0.0843524	0.9599361

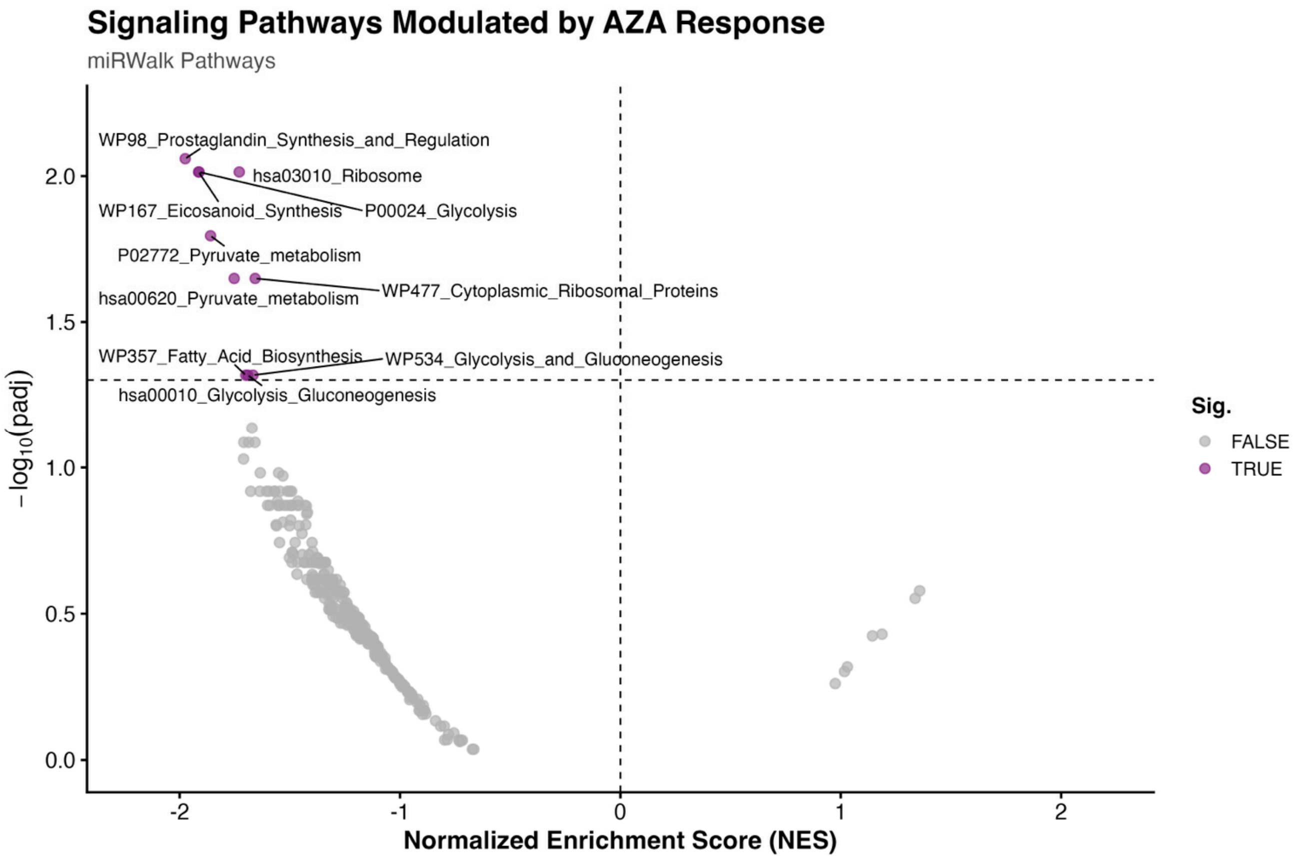

Figure S2. Signaling Pathways Modulated by AZA Response (miRWalk)

Volcano plot displaying signaling pathways enriched in the AZA response analysis using the miRWalk database. Significant pathways (purple) with negative normalized enrichment scores (NES) include Prostaglandin Synthesis and Regulation, Ribosome, Glycolysis, and Pyruvate Metabolism.

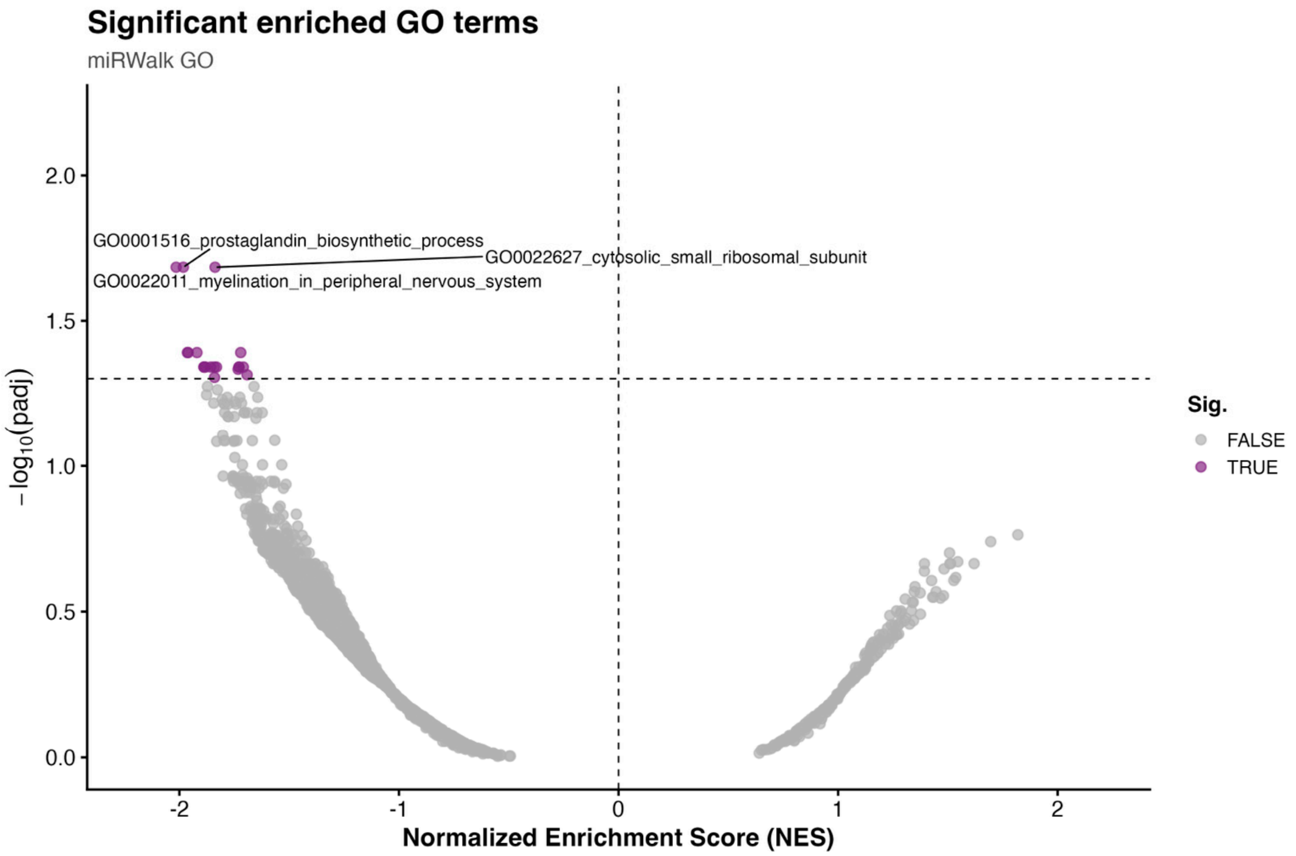

Figure S3. Significant Enriched GO Terms (miRWalk)
Volcano plot showing Gene Ontology (GO) terms significantly enriched in the AZA response using miRWalk. database. Top significant terms (negative NES) include prostaglandin biosynthetic process, cytosolic small ribosomal subunit, and myelination in the peripheral nervous system.

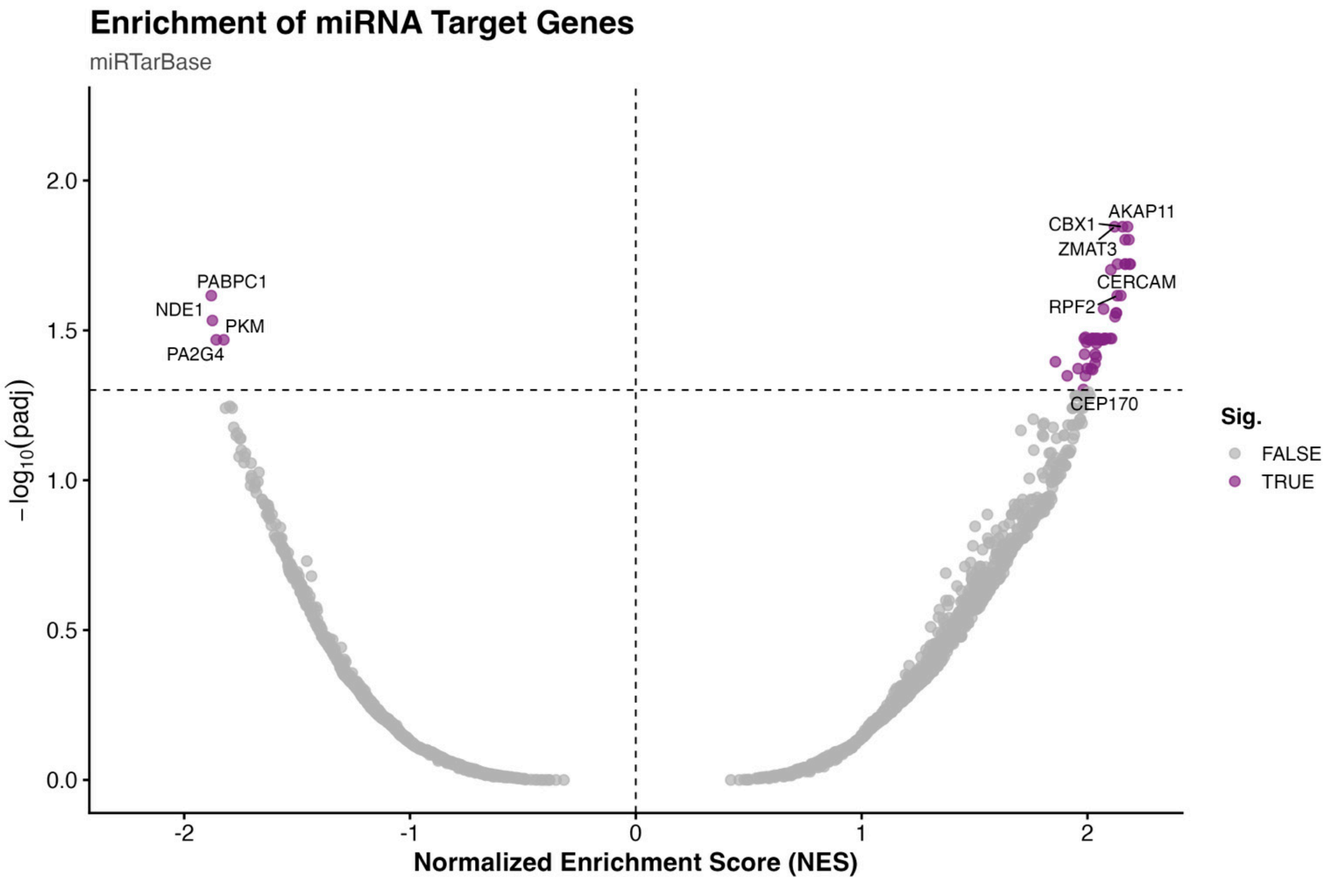

Figure S4. Enrichment of miRNA Target Genes (miRTarBase)
Volcano plot illustrating the enrichment of specific miRNA target genes validated by miRTarBase. Significant targets with positive NES include AKAP11, CBX1, and ZMAT3, while targets like PABPC1, NDE1, and PKM show negative NES.

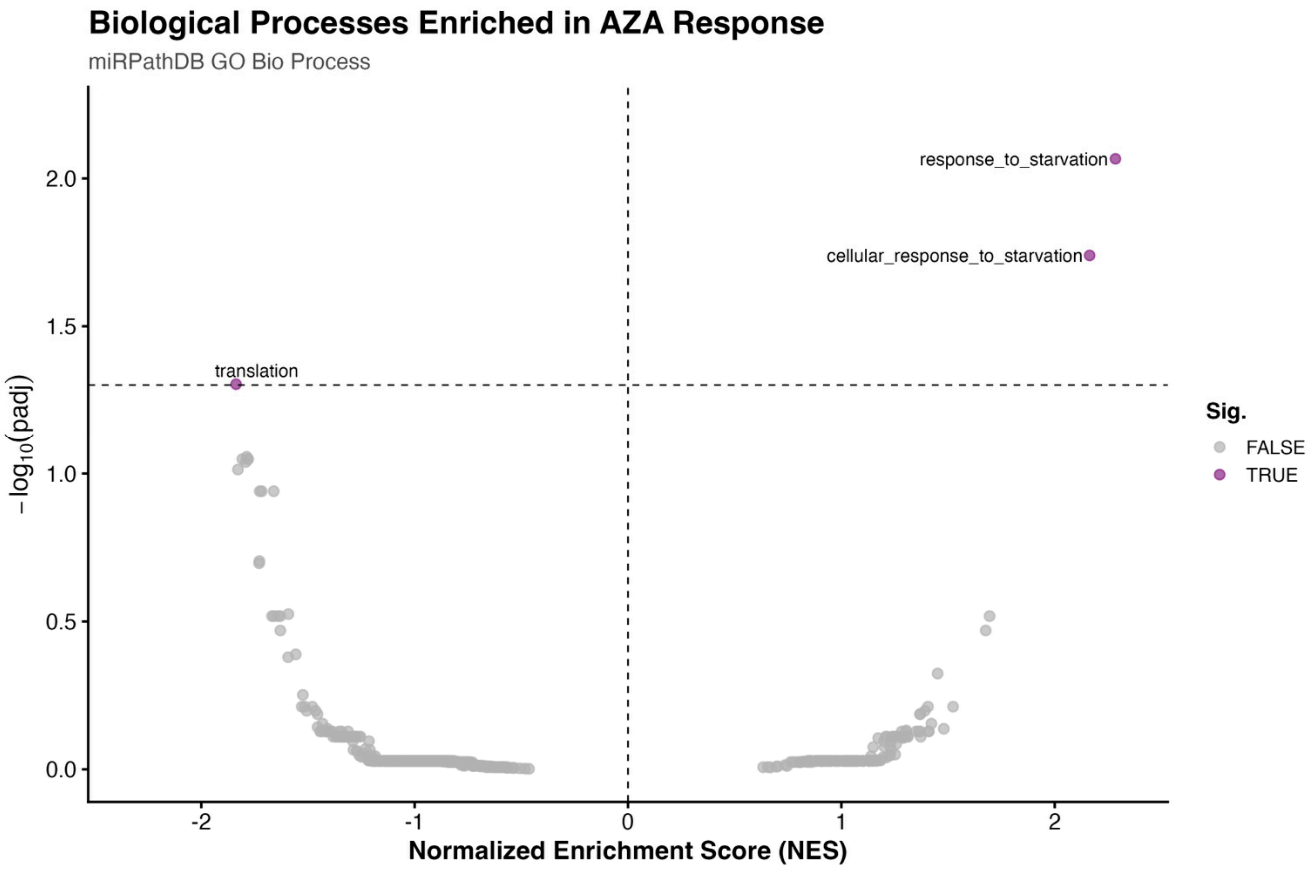

Figure S5. Biological Processes Enriched in AZA Response (miRPathDB)
Volcano plot of biological processes identified via miRPathDB database. "Response to starvation" and "cellular response to starvation" show positive NES enrichment, while "translation" shows negative NES enrichment.

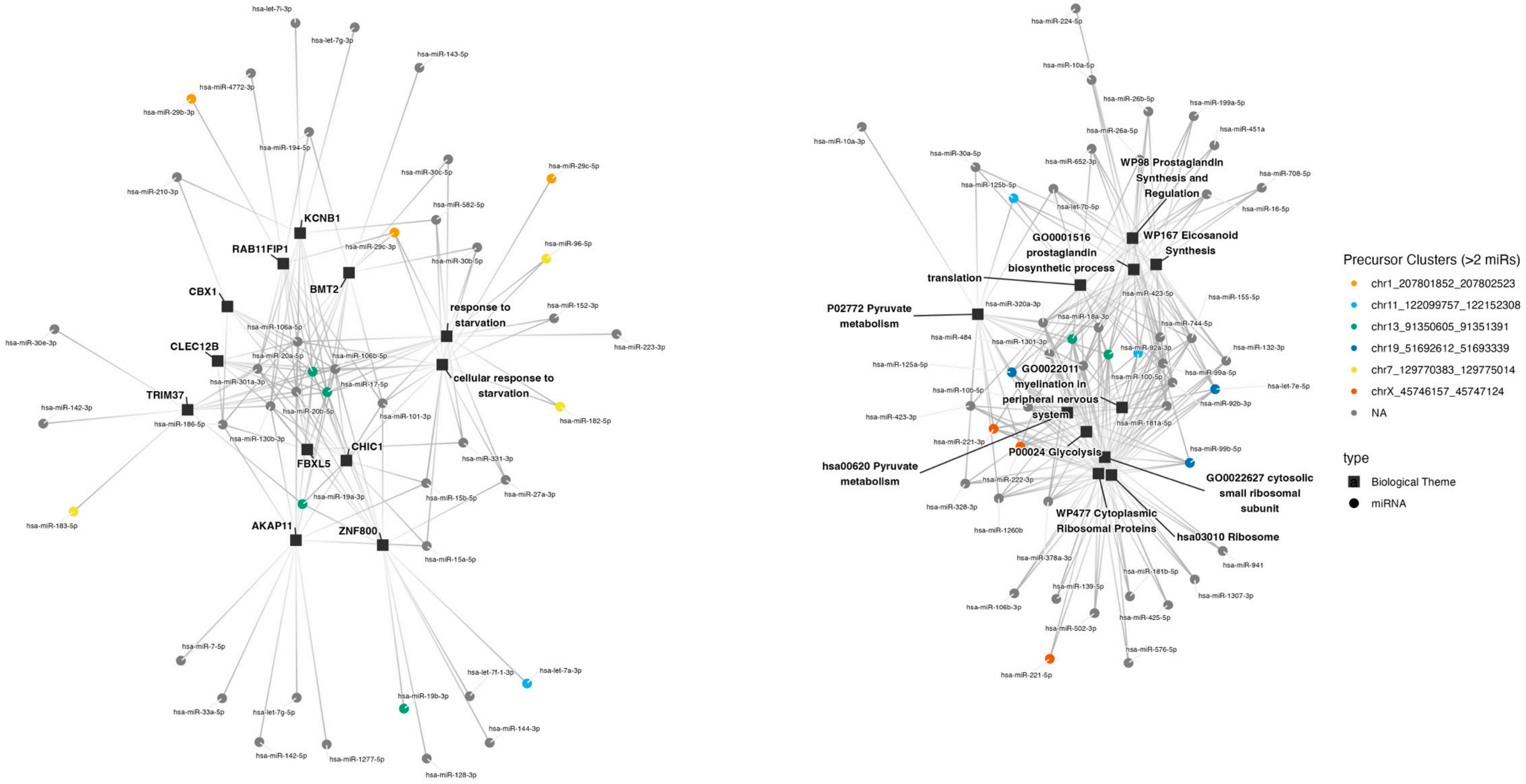

Figure S6. Network Analysis of miRNA Clusters and Targets
Network diagram linking regulatory miRNAs (circular nodes) to biological themes (square nodes). This visualization uses the same topology as Figure 1D but nodes are colored by Precursor Clusters that contain more than 2 miRNAs displayed in the Network.

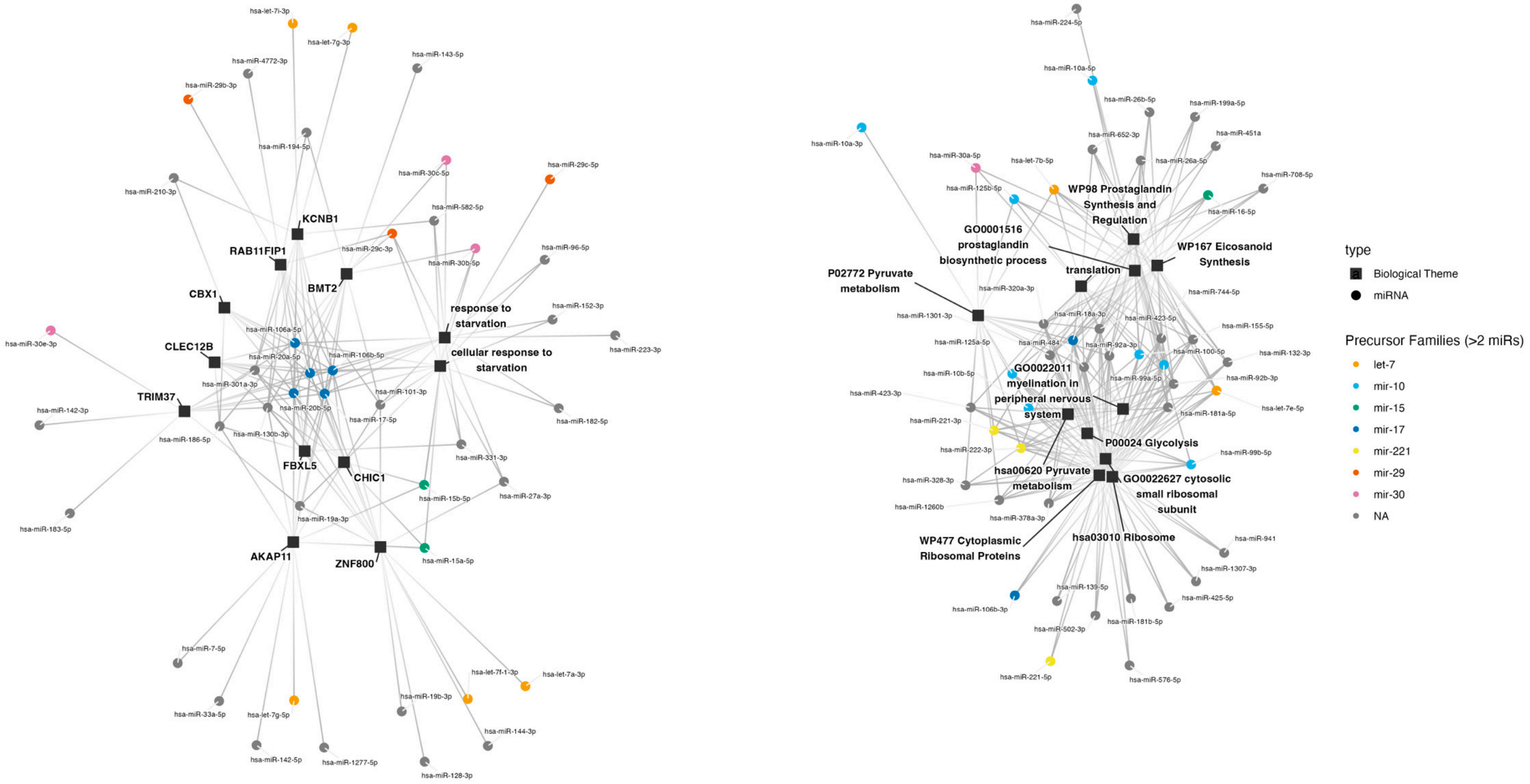

Figure S7. Network Analysis of miRNA Families and Targets

Network diagram linking regulatory miRNAs (circular nodes) to biological themes (square nodes). This visualization uses the same topology as Figure 1D but nodes are colored by Precursor Families that contain more than 2 miRNAs displayed in the Network.

LOD, LOQ and RSD evaluations of LC/MS-MS methodology utilized for RNA modification estimations

LOD and LOQ were estimated from the calibration curves according to the ICH Q2(R2) guideline as LOD = 3.3 × (σ/S) and LOQ = 10 × (σ/S), where S is the slope of the calibration curve and σ the standard deviation of the y-intercept. For each RNA modification these were determined over the working concentration range bracketing the endogenous levels of the samples and all quantified values fell above the LOQ (**Supplementary Table ST4**). Intra-day precision was assessed from same-day duplicate injections of each sample and expressed as %RSD which was consistently low across all five modifications (overall mean 3.9% with all pairs below the 15% acceptance criterion (**Supplementary Table ST2** and **Supplementary Table ST3**).

Supplementary Table S3. LOD and LOQ for each RNA modification (LC-MS/MS).

Analytical Sensitivity: Working Range, LOD, and LOQ Parameters

Target Modification	Working Range (ppb)	LOD (ppb)	LOQ (ppb)
m5C	0.5–5	0.26	0.80
m6A	0.2–10	0.24	0.73
Gm	0.5–20	0.71	2.16
Inosine	5–500	7.37	22.32
m1A	0.1–100	0.31	0.93

Supplementary Table S4. Intra-day precision (%RSD) per RNA modification.

Method Repeatability Summary by Modification Type

Target Modification	n (pairs)	Mean RSD (%)	Range RSD (%)
m5C	16	4.39	0.97–8.63
m6A	16	5.19	1.15–9.46
Gm	16	3.03	0.60–5.96
Inosine	16	4.07	0.26–9.28
m1A	16	2.99	0.80–5.12
**Overall**	**80**	**3.94**	**0.26–9.46**

Supplementary Table S5. Intra-day precision (repeatability) for RNA modification measurements (duplicate injections per sample).

RNA Modification and Repeatability Analysis

Patient ID	Response	Timepoint	Modification	Run 1 (ppb)	Run 2 (ppb)	Mean (ppb)	RSD (%)
NR4-PRE	Non-Responder	Pre	5mC	1.92	1.79	1.86	4.96
NR4-POST	Non-Responder	Post	5mC	1.85	1.98	1.92	4.80
NR2-PRE	Non-Responder	Pre	5mC	2.00	2.26	2.13	8.63
NR2-POST	Non-Responder	Post	5mC	1.41	1.32	1.37	4.66
NR3-PRE	Non-Responder	Pre	5mC	2.60	2.82	2.71	5.74
NR3-POST	Non-Responder	Post	5mC	2.81	2.53	2.67	7.42
NR1-PRE	Non-Responder	Pre	5mC	2.17	2.20	2.19	0.97
NR1-POST	Non-Responder	Post	5mC	1.82	1.94	1.88	4.51
R4-PRE	Responder	Pre	5mC	2.19	2.03	2.11	5.36
R4-POST	Responder	Post	5mC	68.77	70.00	69.39	1.25
R2-PRE	Responder	Pre	5mC	1.90	2.07	1.99	6.06
R2-POST	Responder	Post	5mC	1.66	1.75	1.71	3.73
R1-PRE	Responder	Pre	5mC	2.03	2.09	2.06	2.06
R1-POST	Responder	Post	5mC	2.30	2.22	2.26	2.50
R3-PRE	Responder	Pre	5mC	1.87	2.04	1.96	6.15
R3-POST	Responder	Post	5mC	0.95	0.97	0.96	1.47
NR4-PRE	Non-Responder	Pre	m6A	5.66	6.28	5.97	7.34
NR4-POST	Non-Responder	Post	m6A	5.43	6.01	5.72	7.17
NR2-PRE	Non-Responder	Pre	m6A	5.30	6.06	5.68	9.46
NR2-POST	Non-Responder	Post	m6A	24.88	24.48	24.68	1.15
NR3-PRE	Non-Responder	Pre	m6A	3.80	3.46	3.63	6.62
NR3-POST	Non-Responder	Post	m6A	5.30	4.67	4.99	8.94
NR1-PRE	Non-Responder	Pre	m6A	6.05	6.38	6.22	3.75
NR1-POST	Non-Responder	Post	m6A	4.41	4.85	4.63	6.72
R4-PRE	Responder	Pre	m6A	7.54	7.94	7.74	3.65
R4-POST	Responder	Post	m6A	3.70	3.46	3.58	4.74
R2-PRE	Responder	Pre	m6A	4.40	4.26	4.33	2.29
R2-POST	Responder	Post	m6A	5.32	5.10	5.21	2.99
R1-PRE	Responder	Pre	m6A	6.10	6.42	6.26	3.61
R1-POST	Responder	Post	m6A	5.39	5.74	5.57	4.45

Patient ID	Response	Timepoint	Modification	Run 1 (ppb)	Run 2 (ppb)	Mean (ppb)	RSD (%)
R3-PRE	Responder	Pre	m6A	5.98	6.26	6.12	3.24
R3-POST	Responder	Post	m6A	2.22	2.45	2.34	6.97
NR4-PRE	Non-Responder	Pre	Gm	10.06	9.69	9.88	2.65
NR4-POST	Non-Responder	Post	Gm	9.09	9.68	9.39	4.45
NR2-PRE	Non-Responder	Pre	Gm	9.00	8.83	8.92	1.35
NR2-POST	Non-Responder	Post	Gm	12.16	11.94	12.05	1.29
NR3-PRE	Non-Responder	Pre	Gm	4.12	4.30	4.21	3.02
NR3-POST	Non-Responder	Post	Gm	8.34	8.76	8.55	3.47
NR1-PRE	Non-Responder	Pre	Gm	8.12	7.85	7.99	2.39
NR1-POST	Non-Responder	Post	Gm	7.48	6.89	7.19	5.81
R4-PRE	Responder	Pre	Gm	10.09	10.22	10.16	0.91
R4-POST	Responder	Post	Gm	5.13	5.34	5.24	2.84
R2-PRE	Responder	Pre	Gm	5.89	5.84	5.87	0.60
R2-POST	Responder	Post	Gm	6.76	6.98	6.87	2.26
R1-PRE	Responder	Pre	Gm	9.08	9.28	9.18	1.54
R1-POST	Responder	Post	Gm	7.42	6.82	7.12	5.96
R3-PRE	Responder	Pre	Gm	7.39	7.96	7.68	5.25
R3-POST	Responder	Post	Gm	2.93	3.13	3.03	4.67
NR4-PRE	Non-Responder	Pre	Inosine	87.82	92.03	89.93	3.31
NR4-POST	Non-Responder	Post	Inosine	90.60	82.90	86.75	6.28
NR2-PRE	Non-Responder	Pre	Inosine	108.01	118.44	113.23	6.51
NR2-POST	Non-Responder	Post	Inosine	109.56	111.62	110.59	1.32
NR3-PRE	Non-Responder	Pre	Inosine	96.86	96.50	96.68	0.26
NR3-POST	Non-Responder	Post	Inosine	85.56	92.20	88.88	5.28
NR1-PRE	Non-Responder	Pre	Inosine	92.81	95.45	94.13	1.98
NR1-POST	Non-Responder	Post	Inosine	97.75	102.13	99.94	3.10
R4-PRE	Responder	Pre	Inosine	117.23	122.48	119.86	3.10
R4-POST	Responder	Post	Inosine	182.47	190.49	186.48	3.04
R2-PRE	Responder	Pre	Inosine	306.40	292.34	299.37	3.32
R2-POST	Responder	Post	Inosine	81.94	87.47	84.71	4.62
R1-PRE	Responder	Pre	Inosine	89.83	102.45	96.14	9.28

Patient ID	Response	Timepoint	Modification	Run 1 (ppb)	Run 2 (ppb)	Mean (ppb)	RSD (%)
R1-POST	Responder	Post	Inosine	86.11	89.90	88.01	3.05
R3-PRE	Responder	Pre	Inosine	80.43	87.19	83.81	5.70
R3-POST	Responder	Post	Inosine	52.91	56.78	54.85	4.99
NR4-PRE	Non-Responder	Pre	m1A	4.40	4.18	4.29	3.63
NR4-POST	Non-Responder	Post	m1A	4.36	4.24	4.30	1.97
NR2-PRE	Non-Responder	Pre	m1A	4.30	4.54	4.42	3.84
NR2-POST	Non-Responder	Post	m1A	2.41	2.58	2.50	4.82
NR3-PRE	Non-Responder	Pre	m1A	6.56	6.77	6.67	2.23
NR3-POST	Non-Responder	Post	m1A	6.45	6.23	6.34	2.45
NR1-PRE	Non-Responder	Pre	m1A	4.98	5.05	5.02	0.99
NR1-POST	Non-Responder	Post	m1A	3.62	3.70	3.66	1.55
R4-PRE	Responder	Pre	m1A	5.94	5.56	5.75	4.67
R4-POST	Responder	Post	m1A	2.53	2.72	2.63	5.12
R2-PRE	Responder	Pre	m1A	4.26	4.10	4.18	2.71
R2-POST	Responder	Post	m1A	4.92	4.64	4.78	4.14
R1-PRE	Responder	Pre	m1A	4.39	4.44	4.42	0.80
R1-POST	Responder	Post	m1A	4.48	4.28	4.38	3.23
R3-PRE	Responder	Pre	m1A	4.27	4.42	4.35	2.44
R3-POST	Responder	Post	m1A	1.31	1.25	1.28	3.31

A

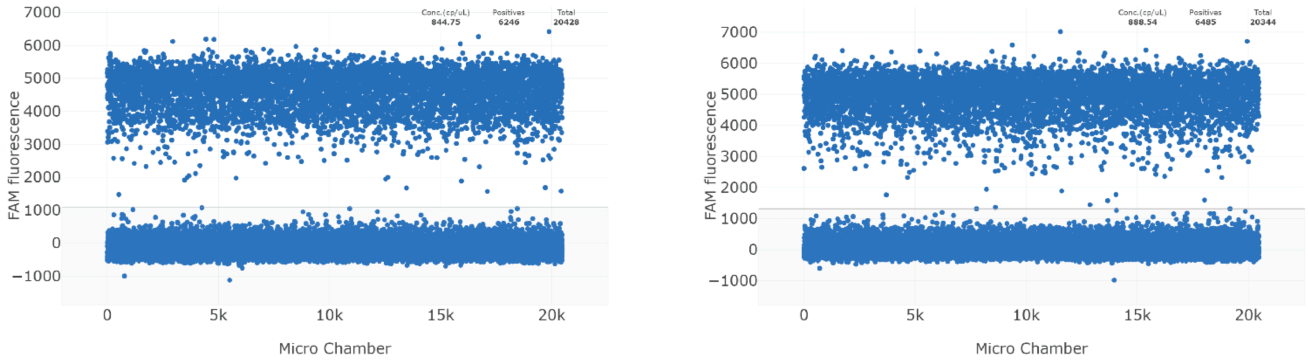

B

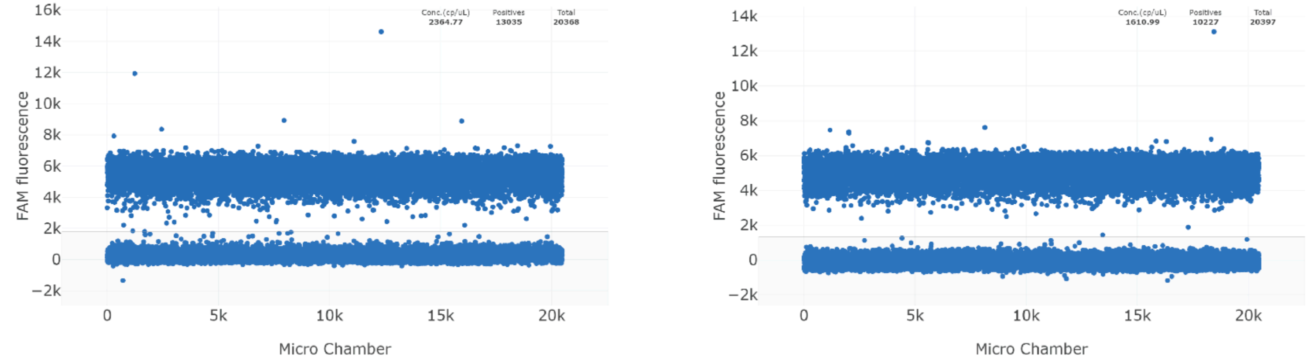

Figure S8. Digital PCR (dPCR) scatter plots for target genes GAPDH and MT-CYB of HR-MDS DNA sample pre- and post-AZA treatment. Unprocessed scatter plots are exported from the QuantStudio Absolute Q dPCR software. x-axis corresponds to the microchamber number, and the y-axis represents the fluorescence intensity (FAM channel). Positive microchambers (containing the target molecule) are distinguished by their increased fluorescence amplitude relative to negative microchambers. **A.** Measurements for the nuclear reference gene GAPDH pre- (left) and post (right)-AZA treatment **B.** Measurements for the mitochondrial gene MT-CYB pre- (left) and post (right)-AZA treatment (all plots derive from patient sample R4)

A

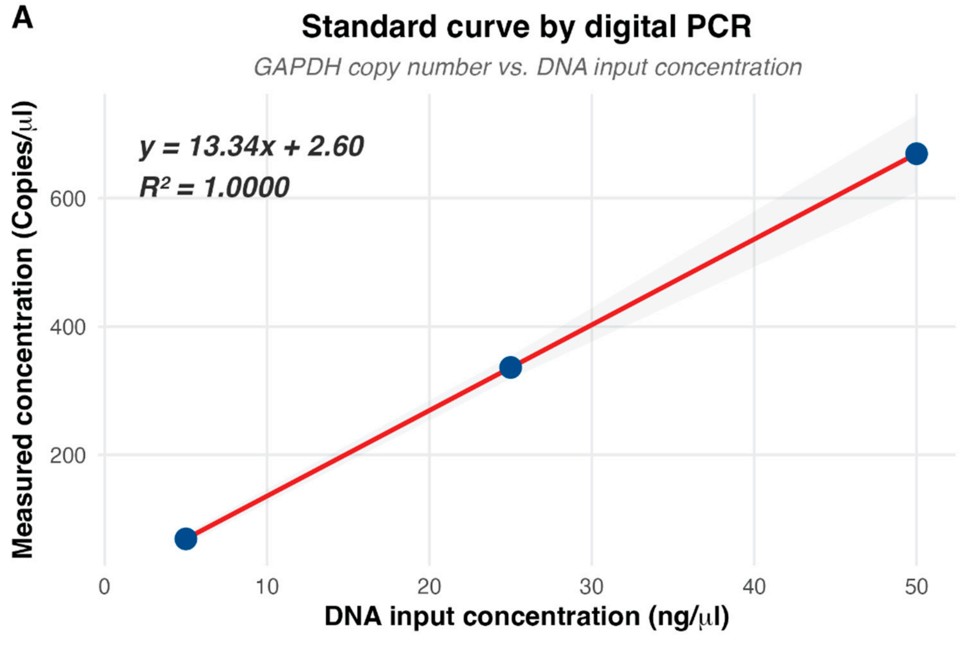

C

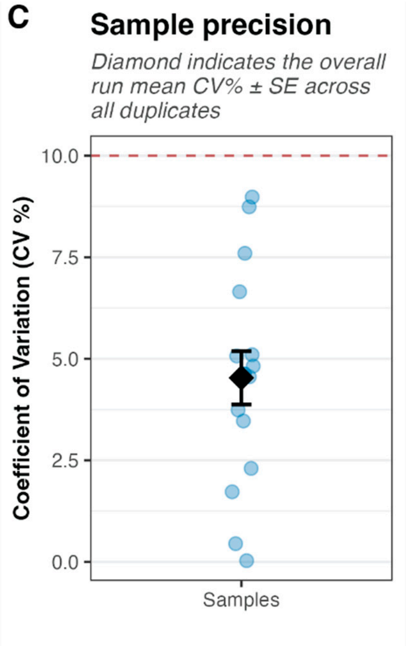

B

Digital PCR Precision and Repeatability Analysis

Target Concentration	Average copies/µL	SD	CV (%)	Average λ
5	69.13	8.44	12.21	0.03
25	336.23	17.27	5.14	0.15
50	669.24	59.85	8.94	0.29

Figure S9. Assessment of the repeatability and linearity of the digital PCR (dPCR) assay. A. Serial dilutions of total DNA (50, 25 and 5 ng) were analyzed in triplicate by digital PCR targeting the *GAPDH* gene to evaluate the repeatability and quantitative performance of the assay. The standard curve demonstrates the linear relationship between DNA input concentration and the measured copy number (copies/µL). **B.** The table summarizes the mean measured concentration (copies/µL), standard deviation (SD), coefficient of variation (CV%), and lambda parameter (λ) for each DNA input concentration. A CV value below 10% was predefined as the acceptance criterion for assay repeatability, while λ values were monitored to ensure appropriate partition occupancy and reliable absolute quantification. **C.** The overall assay precision across all analyzed patient samples is presented as the mean CV% ± SE, with a global assay precision of 4.53%, further supporting the reproducibility of the dPCR assay.
